# Atomically Dispersed Zr‐N Moieties Modulate Fe Coordination for Robust Oxygen Reduction Electrocatalysis

**DOI:** 10.1002/advs.202512381

**Published:** 2025-09-29

**Authors:** Siqi Qiu, Hao Wan, Yuechao Yao, Xiao Xu, Zhangjian Li, Yongbiao Mu, Biaolin Peng, Hongliang Wu, Jizhao Zou, Lin Zeng

**Affiliations:** ^1^ Guangdong Provincial Key Laboratory of New Energy Materials Service Safety & Shenzhen Key Laboratory of Special Functional Materials & Shenzhen Engineering Laboratory for Advance Technology of Ceramics College of Materials Science and Engineering Shenzhen University Shenzhen Guangdong 518060 P. R. China; ^2^ Fritz‐haber‐institut der max‐planck‐gesellschaft 14195 Berlin Germany; ^3^ Department of Mechanical and Energy Engineering Southern University of Science and Technology Shenzhen Guangdong 518060 P. R. China; ^4^ School of Advanced Materials and Nanotechnology Xidian University Xi'an 710126 P. R. China

**Keywords:** dual‐atom catalysts, Fe coordination environment, oxygen reduction reaction, zinc–air batteries

## Abstract

Iron‐nitrogen‐carbon (Fe‐N‐C) materials are promising non‐precious metal catalysts for the oxygen reduction reaction (ORR), yet their long‐term stability remains a critical challenge due to the dissolution of Fe‐based active sites and corrosion of the carbon support. Here, a Fe‐Zr dual‐atom carbon‐based catalyst (Fe,Zr‐NC) via a one‐step solid‐state synthesis method is reported. The introduction of atomically dispersed Zr–N units adjacent to Fe–N_4_ centers creates a dual‐metal coordination structure that modulates the local electronic environment of Fe and weakens ^*^OH adsorption, which is the rate‐limiting step in the ORR process. Density functional theory (DFT) calculations reveal that the Fe–Zr synergy positions the catalyst near the apex of the ORR activity volcano. Extended experimental EXAFS confirms a Fe–Zr distance of ≈3.30 Å, closely matching the theoretical optimum (≈3.39 Å). As a result, Fe, Zr‐NC achieves a high half‐wave potential (0.891 V vs reversible hydrogen electrode (RHE)) with negligible activity loss over 5000 cyclic voltammetry cycles, outperforming commercial Pt/C. In zinc–air batteries, the catalyst delivers a peak power density of 185.7 mW cm^−2^ and operates stably for over 453 h. This work highlights the importance of dual‐atom synergy in tuning intermediate binding energies and provides design principles for next‐generation ORR electrocatalysts.

## Graphical abstract

The introduction of atomically dispersed Zr‐N moieties adjacent to Fe‐N_4_ centers forms a bimetallic coordination configuration. This structure modifies the local electronic environment of the Fe sites, weakening the adsorption of OH intermediates, suppressing Fe agglomeration and dissolution, and consequently enhancing both the catalytic activity and stability for the ORR.

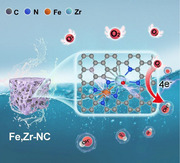

## 1 Introduction

The escalating global energy demand, coupled with the depletion of fossil fuel reserves and worsening environmental degradation,^[^ ^1^ ^]^ underscores the urgent need to explore renewable energy sources and develop efficient energy storage and conversion devices. The oxygen reduction reaction (ORR), a critical process in full cells and metal‐air batteries, suffers from sluggish kinetics due to the complex multi‐electron transfer pathway.^[^ ^2^ ^]^ While platinum‐based catalysts remain the benchmark, their high cost, scarcity, and limited long‐term durability impede widespread commercialization.^[^ ^3^ ^]^ As a result, the development of highly efficient non‐precious metal catalysts has become imperative. Among various alternatives,^[^ ^4^ ^]^ Fe‐N‐C catalysts have emerged as leading ORR candidates owing to their high catalytic activity and selectivity toward the four‐electron ORR pathway.^[^ ^5^ ^]^ Their performance stems from atomically dispersed Fe‐N_X_ moieties embedded in nitrogen‐doped carbon, which enhance both electron transfer and intermediate adsorption.^[^ ^4^, ^6^ ^]^ However, the practical application of such catalysts is severely limited by poor stability, as peroxide species generated via the two‐electron pathway can attack both the Fe active centers and the carbon matrix, leading to metal leaching and carbon corrosion, ultimately causing rapid performance decay.^[^ ^3^, ^7^ ^]^ Although Fe–N–C catalysts exhibit outstanding oxygen reduction reaction activity, the accumulation of reaction intermediates renders stability a critical bottleneck.

To address the limitations, incorporating secondary metal elements, such as cobalt,^[^ ^8^ ^]^ nickel,^[^ ^9^ ^]^ or copper^[^ ^10^ ^]^ into the Fe‐N_x_‐C framework has been proposed as an effective strategy to tune the Fe coordination environment and enhance catalytic performance. These dual‐atom configurations introduce local asymmetry that modulates electronic structure, optimizes intermediate adsorption, and enhances activity.^[^ ^11^ ^]^ This electronic configuration enables strong electronic interactions with Fe atoms, enhancing ORR activity.^[^ ^12^ ^]^ Unlike Co, Ni, and Cu transitional metal elements, zirconium was selected as the unique metal in the Fe‐based dual‐atom catalyst due to its unique chemical and electronic properties. Its high chemical stability and low solubility suppress H_2_O_2_ formation and prevent metal leaching, enhancing durability.^[^ ^13^ ^]^ As a 4*d* metal with fewer d‐electrons and more delocalized orbitals than typical 3*d* metals, Zr effectively modulates the electronic structure of neighboring Fe–N_4_ sites, mitigating *OH poisoning. In addition, a larger ionic radius and strong nitrogen affinity of Zr facilitate stable Zr–N coordination, further improving structural stability.^[^ ^12b^ ^]^ These features distinguish the Fe–Zr system from conventional 3*d–*3*d* dual‐atom catalysts, highlighting advantages for the design of efficient ORR catalysts. These findings collectively support the view that Fe–Zr synergy is a viable strategy for developing high‐performance, durable electrocatalysts. Despite these advantages, most reported Fe, Zr dual‐atom catalysts involve complex synthesis procedures,^[^ ^7^, ^14^ ^]^ which further hinder their facile production and practical application. Moreover, the deactivation mechanisms of Fe, Zr dual‐atom catalysts during extended operation remain poorly understood. Therefore, developing a simple, scalable, and cost‐effective method to fabricate robust Fe, Zr dual‐atom catalysts—while uncovering the structure–activity–durability relationship—is of both fundamental and practical significance.

In this work, a highly active and durable Fe,Zr‐NC electrocatalyst with hierarchical porosity and atomically dispersed active sites was successfully synthesized through a rapid thermal expansion process followed by carbonization. The resulting catalyst exhibits excellent ORR performance in alkaline media, delivering a high half‐wave potential (*E*_1/2_) of 0.891 V vs. RHE and superior durability over 5500 cycles. X‐ray absorption spectroscopy (XAS) reveals an asymmetric Fe‐Zr‐N_3_ and Zr‐N_4_ configurations, while EXAFS confirms a Fe–Zr distance (≈3.30 Å) in close agreement with the DFT‐predicted optimum (≈3.39 Å). Theoretical analysis demonstrates that Zr facilitates ^*^OH desorption, modulates the Fe electronic structure, and optimizes the adsorption of key intermediates, placing the catalyst near the apex of the ORR activity volcano. When applied as an air cathode in zinc–air batteries, the catalyst achieves a peak power density of 185.7 mW cm^−2^ and operates stably for over 453 h. Moreover, combined XPS, electrochemical, and HRTEM analyses uncover the deactivation mechanism, offering deep insights into performance degradation. This work not only demonstrates a scalable route to fabricate robust dual‐atom catalysts but also provides mechanistic insights into Fe–Zr synergy, offering guidance for the rational design of advanced ORR electrocatalysts.

## 2 Results and Discussion

### 2.1 Materials Synthesis and Characterization

**Figure 1:**
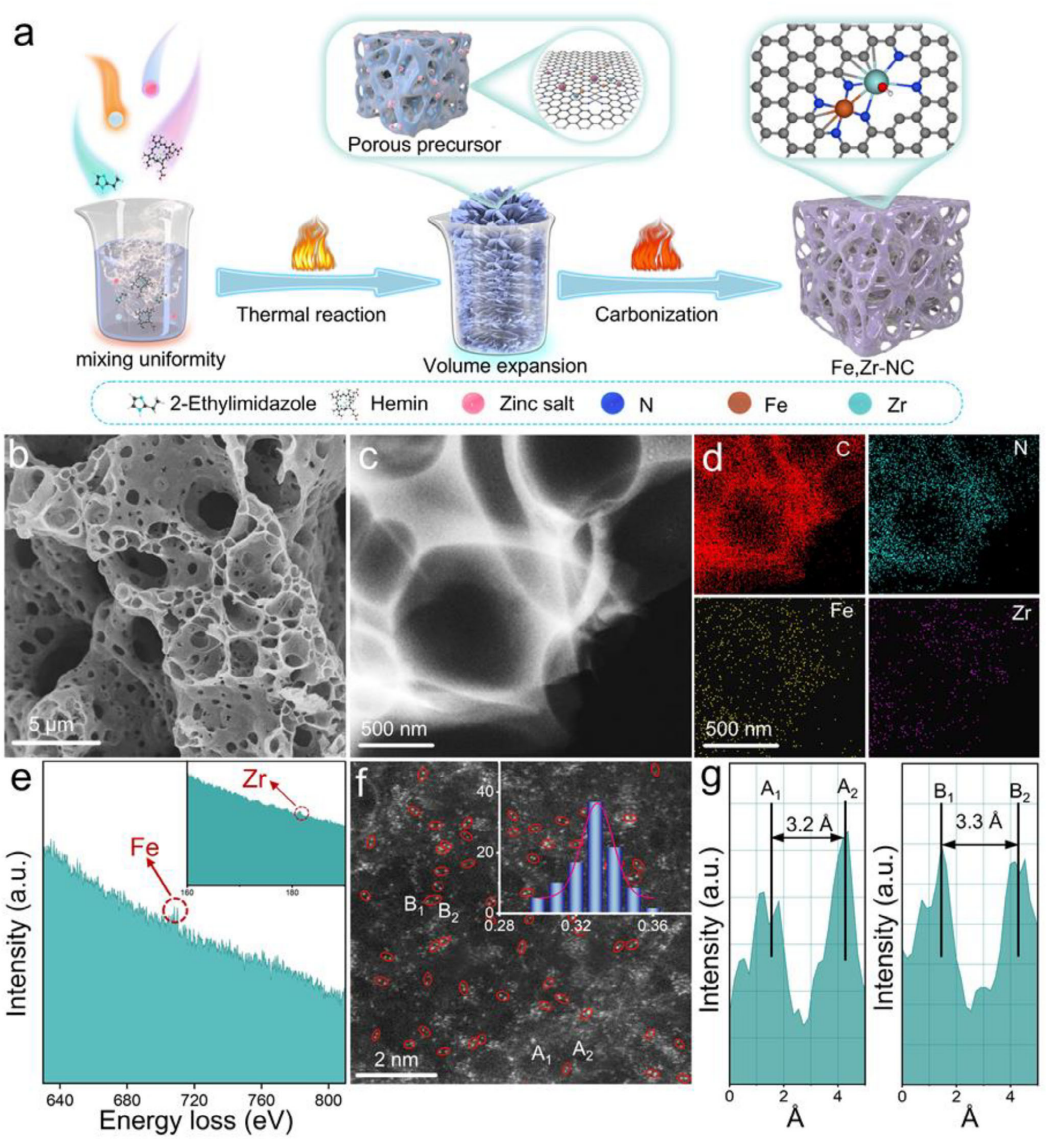
Synthesis process and structural characterization of Fe,Zr‐NC. a) Schematic illustration for the preparation process of Fe,Zr‐NC. b) SEM images of Fe, Zr‐NC, c) TEM images of Fe, Zr‐NC, d) EDS mapping images of C, N, Fe, and Zr elements, e) EELS Fe, Zr‐NC structure analyzed by EELS, f) HAADF‐STEM image of Fe, Zr‐NC (insert: Statistical Fe–Zr distance in the observed diatomic pairs.). g) The intensity profiles obtained on two bimetallic Fe–Zr sites.

**Figure** **1**a illustrates the synthesis process of atomically dispersed M_x_‐N‐C electrocatalysts. A solid‐state thermal reaction between diethyl imidazole and metal salts was conducted at 170 °C, leading to the formation of a porous organic‐carbon precursor (for detailed procedures, see the Experimental Section in the Supporting Information). This reaction induces significant volumetric expansion, as shown in Figure S1 (Supporting Information). The resulting precursor was subsequently carbonized under an argon atmosphere at 950 °C to yield the final electrocatalyst. Scanning electron microscopy (SEM) characterization on the precursor reveals a large number of uniformly distributed particles (Figure S2a,b, Supporting Information), identified as zinc oxides via X‐ray diffraction and high‐resolution TEM lattice fringe analysis (Figure S2c–e, Supporting Information).^[^ ^15^ ^]^ Notably, these uniformly dispersed zinc oxide particles evaporated during carbonization, generating numerous pores. This porous structure with a high specific surface area provides abundant ion‐diffusion pathways, greatly enhancing electrochemical activity.^[^ ^16^ ^]^

SEM images (Figures 1b; S3–S6, Supporting Information) show that the Fe,Zr‐NC, Fe‐NC, Zr‐NC, and NC catalysts maintain a porous nanosheet morphology inherited from the heat‐treated precursor. Transmission electron microscopy (TEM) image of Fe,Zr‐NC (Figure 1c), further confirms the ultrathin layered carbon structure. The Energy Dispersive Spectrometer (EDS) mapping of Fe,Zr‐NC (Figure 1d) demonstrates the uniform distribution of Fe and Zr across the carbon matrix, verifying the successful incorporation of Fe, Zr, N, and C elements. Inductively coupled plasma optical emission spectrometry (ICP‐OES) analysis (Table S1, Supporting Information) confirmed that the Fe and Zr contents were 1.88 and 1.18 wt.%, respectively, which is consistent with the intended synthesis formulation. Meanwhile, the residual zinc is detected and its content was decreased with increasing pyrolysis temperature, measuring 3.55 wt.% at 900 °C, 1.88 wt.% at 950 °C, and 0.03 wt.% at 1000 °C (Table S2, Supporting Information). Additionally, the electron energy loss spectroscopy (EELS) spectrum (Figure 1e) confirms the coexistence of Fe and Zr in the catalyst. Aberration‐corrected high‐angle annular dark‐field scanning TEM (HAADF‐STEM) image (Figure 1f) clearly reveals atomic dispersion of Fe and Zr species. Red circles highlight Fe and Zr atoms located in proximity, while other visible atomic sites confirm the individual dispersion of metal atoms. Statistical analysis of the Fe–Zr interatomic distances (inset, Figure 1f) shows a predominant spacing of ≈3.3 Å, exhibiting a pyramidal distribution. Atomic spacing measurements in regions A and B (Figure 1g) show Fe–Zr distances of ≈3.2 and 3.3 Å, respectively.

The carbon structure of Fe,Zr‐NC was further examined by X‐ray diffraction (XRD). As shown in Figure S7 (Supporting Information), all the catalysts (Fe,Zr‐NC, Fe‐NC, Zr‐NC, and NC) exhibit similar patterns with two broad diffraction peaks centered at ∼24° and ∼44°, corresponding to the (002) and (101) planes of graphitic carbon, respectively. The absence of diffraction peaks corresponding to metal crystals or clusters confirms the atomic dispersion of metal species.^[^ ^17^ ^]^ Raman spectroscopy reveals characteristic D and G bands at 1340 and 1586 cm^−1^, respectively (Figure S8, Supporting Information). The D band is attributed to structural defects in the carbon lattice, while the G band corresponds to the in‐plane vibrations of sp^2^‐hybridized carbon atoms. Among all samples, Fe,Zr‐NC shows the highest I_D_/I_G_ ratio (3.01), compared to Fe‐NC (2.79), Zr‐NC (2.55), and NC (2.38). A higher I_D_/I_G_ ratio indicates a greater density of structural defects, which are believed to facilitate the exposure of more catalytically active sites and enhance overall ORR performance.^[^ ^18^ ^]^ In order to further confirm this conclusion, the BET specific surface area test was chosen for characterization. Figure S9 (Supporting Information) presents the N_2_ adsorption‐desorption isotherms of Fe,Zr‐NC, Fe‐NC, and Zr‐NC. The isotherms exhibit typical Type IV behavior, indicative of abundant mesoporous structures.^[^ ^19^ ^]^ The Fe,Zr‐NC catalyst shows the highest specific surface area of 668.82 m^2^g^−1^. As summarized in Table S3 (Supporting Information), all three catalysts display hierarchical pore structure comprising micropores, mesopores, and macropores. Notably, Fe,Zr‐NC has the highest micropore volume, which could facilitate the exposure of active sites. The mesoporous network with larger pore diameters promotes efficient mass transport of reactants and products, reducing diffusion resistance and improving catalytic performance. These results highlight the key role of Zn volatilization during pyrolysis in generating hierarchical porosity and optimizing the structural and electrochemical properties of M_x_‐N‐C electrocatalysts.^[^ ^20^ ^]^

### 2.2 Electronic and Atomic Structure Analysis

**Figure 2:**
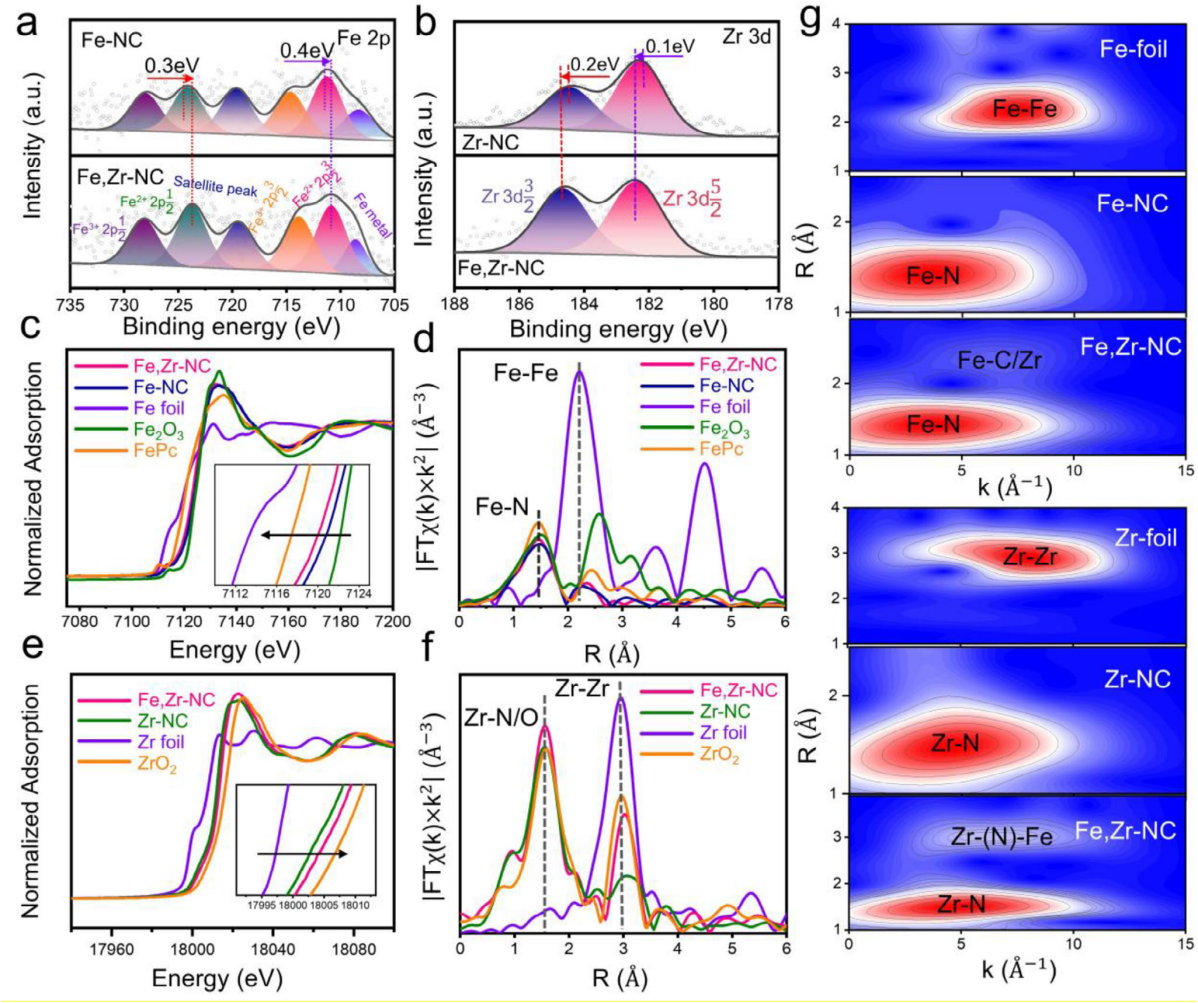
a,b) XPS spectra of Fe 2*p* and Zr 3*d* in catalysts, respectively. c) Fe K‐edge XANES, d) FT‐EXAFS of the Fe K‐edge, e) Zr K‐edge XANES,f) FT‐EXAFS of the Zr K‐edge, g) WT plots of Fe,Zr‐NC in comparison with the Fe foil and Zr foil.

The valence states, elemental composition, and chemical environment of Fe and Zr in the Fe,Zr‐NC catalyst were investigated using X‐ray photoelectron spectroscopy (XPS). The survey XPS spectrum (Figure S10, Supporting Information) confirms the presence of Fe, Zr, N, and C elements in the catalyst. The C 1*s* spectrum is presented in Figure S11 (Supporting Information). As shown in Figure S12 (Supporting Information), the N 1*s* spectrum was deconvoluted into five distinct peaks, corresponding to oxidized nitrogen (402.3 eV), graphitic nitrogen (401.29 eV), pyrrolic nitrogen (400.59 eV), Fe/Zr–N coordination (399.46 eV), and pyridinic nitrogen (398.35 eV). A comparative analysis of nitrogen species between Fe‐NC and Fe,Zr‐NC (Table S4, Supporting Information) reveals notable differences. Specifically, the content of pyrrolic nitrogen and metal‐coordinated nitrogen (M_x_–N, where M = Fe or Zr) in Fe,Zr‐NC reaches 40.6%, slightly higher than the 39.0% in Fe‐NC. More importantly, Fe,Zr‐NC exhibits a significantly higher proportion of pyridinic nitrogen, which is widely recognized as a key catalytic active site. The enrichment of pyridinic nitrogen thus contributes to the superior catalytic performance of the Fe,Zr‐NC catalyst.^[^ ^21^ ^]^ **Figure** **2**a presents the deconvoluted high‐resolution Fe 2*p* XPS spectra for both Fe,Zr‐NC and Fe‐NC. In Fe,Zr‐NC, the binding energies for Fe^2+^2*p*_1/2_ (723.71 eV) and Fe^3+^2*p*_3/2_ (713.81 eV) exhibit a negative shift compared to those in Fe‐NC, indicating a partial reduction of iron species.^[^ ^22^ ^]^ Conversely, the Zr 3*d* peaks in Fe,Zr‐NC (Figure 2b) show a positive shift relative to those in Zr‐NC, implying an increase in the oxidation state of Zr. This charge redistribution suggests that Zr may donate a partial negative charge to Fe, thereby lowering the oxidation state of Fe. The introduction of Zr likely influences the local electronic environment by modulating the distribution of Fe and N species, potentially leading to the formation of interconnected Fe–N and Zr–N structures bridged through nitrogen atoms.^[^ ^23^ ^]^ This electronic interaction alters the adsorption behavior of O_2_ and its reaction intermediates at Fe active sites, optimizing the binding energy between O_2_ and Fe.^[^ ^24^ ^]^ The electronic modulation is crucial in enhancing the oxygen reduction reaction (ORR) catalytic activity of Fe, Zr‐NC.

X‐ray absorption spectroscopy (XAS) was employed to investigate the local chemical environment of Fe‐Zr dual‐atomic sites. As shown in Figure 2c, the Fe K‐edge X‐ray absorption near‐edge structure (XANES) spectra reveal that the absorption edge of Fe in Fe,Zr‐NC shifts toward lower energy relative to that in Fe‐NC, indicating a reduced oxidation state of Fe.^[^ ^25^ ^]^ The corresponding Fourier‐transformed extended X‐ray absorption fine structure (FT‐EXAFS) spectra at the Fe K‐edge (Figure 2d) provide further insight into the local coordination environment. In Fe,Zr‐NC, a prominent peak at 1.44 Å is assigned to the Fe–N bond, while peak at 2.33 Å is mainly attributed to Fe─C bonding at the second shell, which is consistent with the reported Fe‐NC structure.^[^ ^26^ ^]^ However, a noticeable difference in peak position in the range of 2–3 Å between Fe‐NC and Fe,Zr‐NC is observed (Figure S13a, Supporting Information), which means that the introduced Zr at the second shell possibly influences the K edge of Fe in Fe,Zr–NC.^[^ ^18a^ ^]^ In contrast, the Zr K‐edge XANES spectra (Figure 2e) of Fe,Zr‐NC show a distinct positive shift in the absorption edge compared to Zr‐NC, suggesting an increased oxidation state of Zr, consistent with the XPS findings(Figure 2a). The Zr K‐edge FT‐EXAFS spectra of Fe,Zr‐NC (Figure 2f) display two peaks at 1.56 Å and 3.01 Å, corresponding to Zr–N and Zr–(N)–Fe scattering paths, respectively. It is noticeable that the FT spectra at Fe K‐edge and Zr K‐edge exhibit distinct second‐shell peaks at ca. 2.30 and 3.00 Å, respectively. These peak position differences could be attributed to the second shell at the Fe K‐edge arising from Fe–C and Fe–Zr co‐contributions versus Zr K‐edge only from the Zr–(N)–Fe. In comparison, the reference Zr‐NC sample shows only a single dominant peak at 1.53 Å, corresponding to Zr–N coordination. The absence of Fe–Fe and Zr–Zr scattering signals across all spectra confirms the atomic dispersion of Fe and Zr species in Fe,Zr‐NC. To gain deeper insight into the coordination structure of Fe‐Zr dual‐atomic sites, quantitative least‐squares EXAFS fitting was performed (Figures S13–S15 and Tables S5 and S6, Supporting Information). From the fitting results, we preliminarily conclude that the first coordination shell around Fe is Fe–N, while the second shell likely comprises co‐existing Fe–C and Fe–Zr contributions.

To further verify the structure of Fe,Zr‐NC, we employed wavelet transform (WT)‐EXAFS analysis was performed (Figure 2g). Two intensity maxima at ≈3.6 and 6.6 Å^−1^ for Fe in Fe,Zr‐NC correspond to Fe–N and Fe–C/Zr coordination, respectively, while the absence of Fe–Fe (7.4 Å^−1^) and Zr–Zr (8.07 Å^−1^) scattering signals confirms atomic dispersion. WT plots of other standard samples are provided in Figure S16 (Supporting Information) for comparison. Similarly, the WT contour plot of Zr in Fe,Zr‐NC displays a maximum intensity at 4.84 Å^−1^, attributed to Zr–N scattering. The absence of Fe–Fe (7.4 Å^−1^) and Zr–Zr (8.07 Å^−1^) scattering signals further supports the atomic dispersion of Fe and Zr in the Fe,Zr‐NC catalyst, in agreement with the FT‐EXAFS results. Taken together with aberration‐corrected HAADF‐STEM imaging and FT‐EXAFS, these WT‐EXAFS results provide strong evidence for the likely presence of atomically dispersed Fe–Zr diatomic sites in the catalyst.^[^ ^27^ ^]^ In addition, we constructed a plausible model in which an Fe–N_4_ site is coupled to an adjacent Zr–N_4_ structural unit, forming the active structural configuration.

### 2.3 Electrocatalytic Activity for Oxygen Reduction

**Figure 3:**
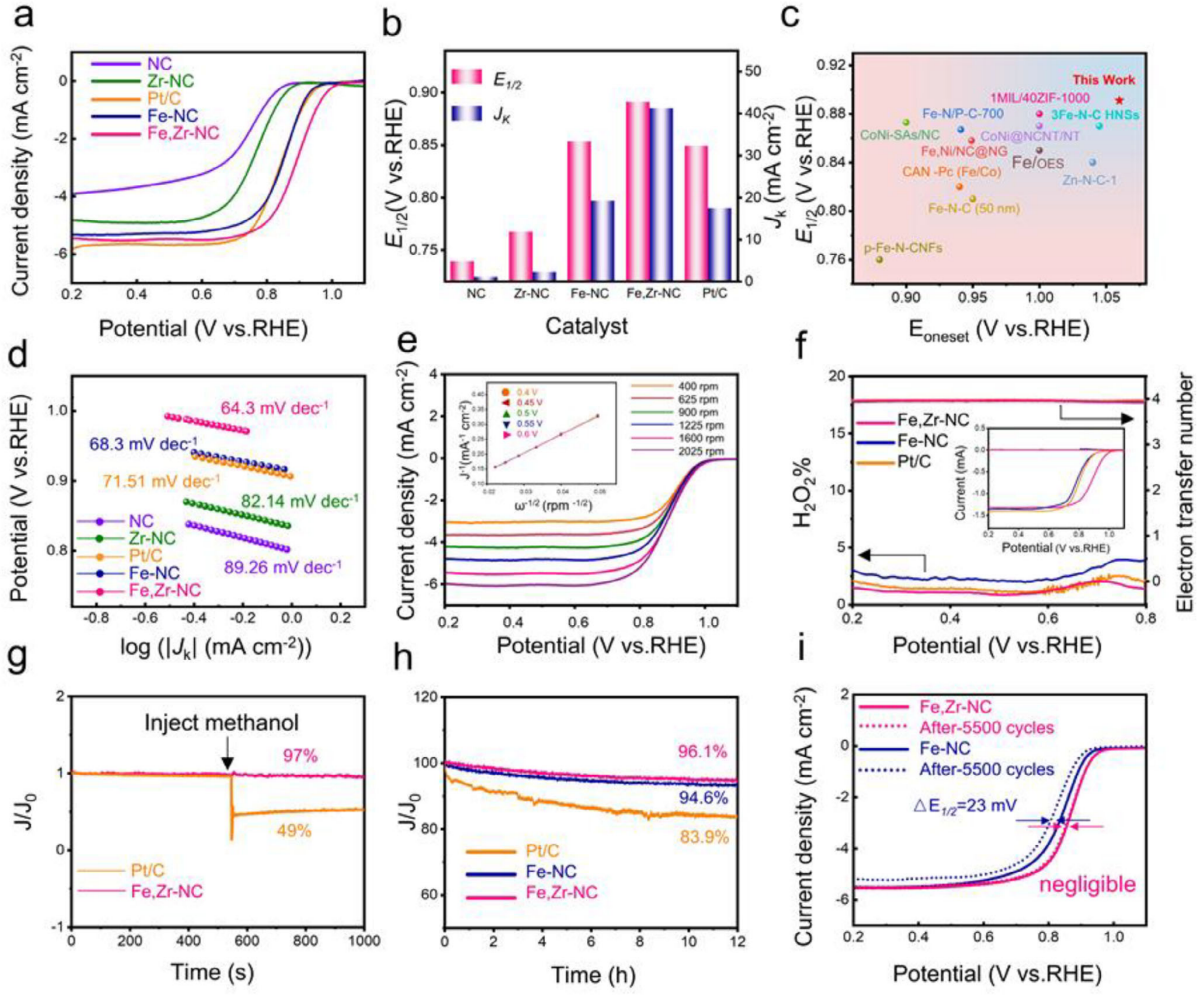
a) LSV curves of Fe, Zr‐NC, Fe‐N‐C, Zr‐N‐C, N‐C, and Pt/C. b) *E*_1/2_ and *J*_k_ at 0.8 V of the catalysts. c) Comparison of *E*_1/2_ and *E*_onset_ in this work with the previously reported catalysts in alkaline solution. d) Transferred Tafel slope from the LSV curves of Fe, Zr‐NC, Fe‐N‐C, Zr‐N‐C, N‐C, and Pt/C. e) LSV curves of Fe,Zr‐NC at different scan rates (inset: corresponding K‐L plots at various electrode potentials). f).H_2_O_2_ yield and electron transfer number from the RRDE measurement of Fe,Zr‐NC, Fe‐NC, and Pt/C. (Insert figures are corresponding RRDE voltammograms recorded in O_2_‐saturated 0.1 m KOH at 1600 rpm). g) methanol resistance test of Fe,Zr‐NC and Pt/C. h) Stability test and i) LSV curves of Fe,Zr‐NC, and Pt/C before and after 5500 CV cycles.

The oxygen reduction reaction (ORR) activity of the catalysts was evaluated in 0.1 m KOH using cyclic voltammetry (CV) and linear sweep voltammetry (LSV) techniques. As shown in Figures S17 and S18 (Supporting Information), the Fe,Zr‐NC catalyst exhibits a pronounced reduction peak at 0.83 V vs. RHE, indicating excellent electrocatalytic performance. The LSV curves of various catalysts (Fe,Zr‐NC, Fe‐NC, Zr‐NC, and N‐C) are compared in **Figure** **3**a. Among them, Fe,Zr‐NC displays the highest catalytic activity with an onset potential (*E*_onset_) of 1.06 V and a half‐wave potential (*E*_1/2_) of 0.891 V, surpassing commercial 20 wt.% Pt/C (*E*onset = 1.00 V, *E*_1/2_ = 0.849 V) as well as Fe‐NC (*E*_onset_ = 1.02 V, *E*_1/2_ = 0.853 V), Zr‐NC (*E*_onset_ = 0.92 V, *E*_1/2_ = 0.767 V), and N‐C (*E*_onset_ = 0.89 V, *E*_1/2_ = 0.739 V). The ORR activity of the Pt/C catalyst is consistent with that previously reported.^[^ ^28^ ^]^ These results confirm that the incorporation of Zr atoms effectively enhances the catalytic activity of Fe‐NC catalysts. Figure 3b compares the kinetic current densities (*J*_k_) at 0.8 V vs. RHE, which serves as an indicator of ORR kinetics. Fe,Zr‐NC achieves the highest *J*_k_ of 48.79 mAcm^−2^, far exceeding Fe‐NC (19.17 mAcm^−2^), Pt/C (17.38 mAcm^−2^), Zr‐NC (2.23 mAcm^−2^), and N‐C (0.96 mAcm^−2^). Figure 3c summarizes the *E*_onset_ and *E*_1/2_ values of various reported non‐precious metal‐based ORR catalysts, revealing that Fe,Zr‐NC demonstrates the highest activity among the listed materials. A broader comparative overview is provided in Table S7 (Supporting Information). Remarkably, this exceptional performance is achieved despite the low catalyst loading of only 0.2 mg cm^−2^. To assess intrinsic catalytic activity, the turnover frequency (TOF) at 0.8 V was calculated based on the assumption that all metal atoms serve as active sites, as is standard for single‐atom catalysts. Fe,Zr‐NC exhibits a TOF of 16.28 s^−1^, the highest among the evaluated samples (Table S8, Supporting Information). The reaction kinetics were further analyzed using Tafel slope measurements (Figure 3d). Fe,Zr‐NC shows a Tafel slope of 64.3 mVdec^−1^, indicative of faster reaction kinetics compared to Fe‐NC (68.3 mV dec^−1^), Zr‐NC (82.14 mV dec^−1^), N‐C (89.26 mV dec^−1^), and Pt/C (71.51 mV dec^−1^), highlighting the synergistic effect between Fe and Zr in accelerating ORR kinetics.

The electron transfer number (n) and H_2_O_2_ yield are key parameters for evaluating ORR selectivity. The Koutecky‐Levich (K‐L) plot (inset, Figure 3e) displays strong linearity and consistent slopes within the potential range of 0.4–0.6 V. For Fe,Zr‐NC, the calculated n values range from 4.00 to 4.06, indicating a nearly ideal four‐electron transfer pathway. Additional comparisons with other catalysts are shown in Figure S19 (Supporting Information). Furthermore, Figure S20 (Supporting Information) clearly indicates the LSV curves of Fe,Zr‐NC and Fe‐NC under acidic conditions, strongly validating the consistent ORR activity of the Fe,Zr‐NC catalyst in acid/alkaline electrolytes. To optimize the Fe:Zr atomic ratio and carbonization temperature, a series of catalysts with Fe:Zr ratios of 1:1 and 2:1 and pyrolysis temperatures ranging from 900 °C to 1000 °C were synthesized. LSV and K‐L plots (Figures S21 and S22, Supporting Information) reveal that the Fe:Zr = 1.5:1 ratio and a carbonization temperature of 950 °C result in the highest catalytic activity.

The four‐electron pathway and low H_2_O_2_ yield of Fe,Zr‐NC were further confirmed through rotating ring‐disk electrode (RRDE) measurements (Figure 3f). The average electron transfer number (n) remained close to 4 over a wide potential window (0.2–0.8 V), with an H_2_O_2_ yield below 3%, demonstrating its high selectivity for efficient ORR. Beyond activity, long‐term durability and methanol tolerance are crucial for practical applications. The methanol tolerance test was conducted by injecting 6 mL of methanol into an O_2_‐saturated 0.1 m KOH electrolyte. As shown in the i‐t curve (Figure 3g), Fe,Zr‐NC maintains a stable current with minimal disturbance, whereas Pt/C exhibits sharp current fluctuations and potential reversal, indicating poor methanol tolerance. Durability was assessed via chronoamperometry and accelerated durability testing (ADT) over 5500 CV cycles. At 0.7 V and 250 rpm, Fe,Zr‐NC retains 96.1% of its initial current after 12 h of continuous operation (Figure 3h), compared to 94.6% for Fe‐NC and 83.9% for Pt/C. Additionally, post i‐t testing analysis shows that the *E*_1/2_ of Fe,Zr‐NC decreased by only 7 mV (Figure S23, Supporting Information), in contrast to 19 mV for Fe‐NC. After 5500 CV cycles (Figures 3I; S24, Supporting Information), Fe,Zr‐NC exhibits a negligible decline in *E*_1/2_, while Fe‐NC and Pt/C show losses of 23 and 38 mV, respectively. Even after 11 500 CV cycles, the *E*_1/2_ of Fe,Zr‐NC decreased by only 8 mV (Figure S25, Supporting Information), confirming its exceptional long‐term electrochemical stability.

### 2.4 Zn–Air Batteries

**Figure 4:**
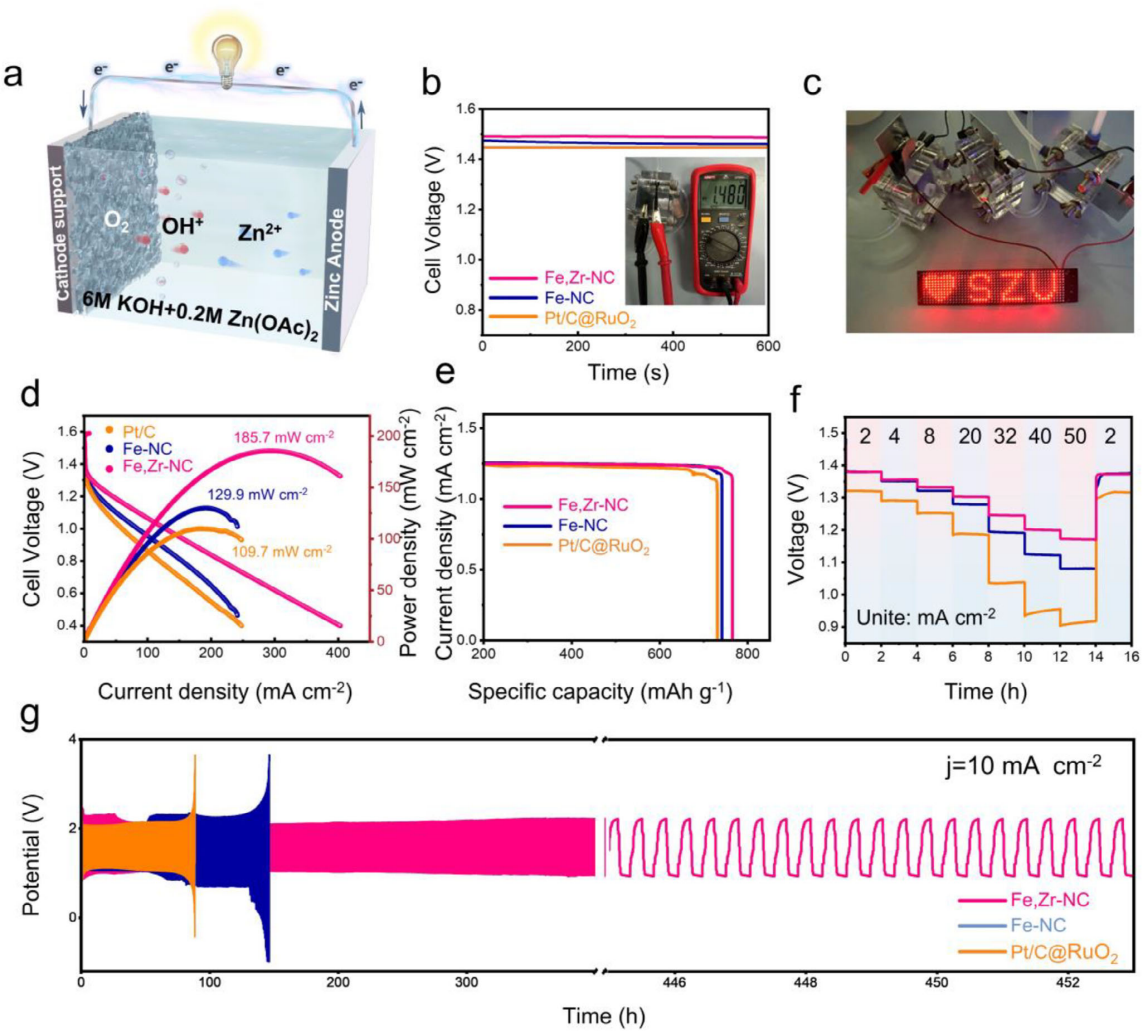
a) Schematic configuration of the assembled rechargeable ZAB. b) Open‐circuit potential of the ZAB based on Fe, Zr‐NC, Fe‐NC, and Pt/C@RuO_2_ (inset: photography of OCP of the ZAB based on Fe, Zr‐NC tested with a multimeter). c) Digital photo of a green LED powered by three series‐connected ZABs based on Fe, Zr‐NC. d) Discharge polarization curves and corresponding power density curves, e) specific capacity, f) galvanostatic discharge curves at various current densities from 2 to 50 mA cm^−2^, and g) galvanostatic discharge/charge cycling performance of the ZAB based on Fe, Zr‐NC, Fe‐NC and Pt/C@RuO_2_.

Given the outstanding ORR performance of Fe,Zr‐NC, this material was further employed as the air electrode catalyst in a self‐assembled aqueous zinc‐air batteries (ZABs) to evaluate its electrocatalytic behavior. The schematic configuration of the ZABs is illustrated in **Figure** **4**a. Fe,Zr‐NC was loaded onto hydrophobic carbon cloth to serve as the air cathode, paired with a polished zinc foil as the anode. The electrolyte comprised an aqueous solution of 6 m KOH and 0.2 m zinc acetate (Zn(Ac)_2_). For comparison, Fe‐NC and commercial 20 wt.% Pt/C@RuO_2_ were also tested under identical conditions as reference cathodes. As shown in Figure 4b, the Fe,Zr‐NC‐based ZABs exhibited an open‐circuit voltage (OCV) of 1.48 V, exceeding those of Fe‐NC (1.46 V) and Pt/C@RuO_2_ (1.45 V). To demonstrate its practical feasibility, three Fe,Zr‐NC‐based ZABs connected in series successfully powered a 3.7 V green LED panel (Figure 4c). The power density curves presented in Figure 4d show that Fe,Zr‐NC achieved a maximum power density of 158.6 mW cm^−2^ at a current density of 254 mA cm^−2^, outperforming Fe‐NC (129.9 mW cm^−2^ at 190 mA cm^−2^) and Pt/C@RuO_2_ (109.7 mW cm^−2^ at 178 mA cm^−2^). Long‐term galvanostatic discharge tests (Figure 4e) estimated a specific capacity of 766 mAhg^−1^ at 20 mA cm^−2^ for Fe,Zr‐NC, which is superior to those of Fe‐NC (742 mAhg^−1^) and Pt/C@RuO_2_ (729 mAhg^−1^). These results underscore the remarkable potential of Fe,Zr‐NC as a promising alternative to noble metal‐based catalysts in zinc–air batteries.

Figure 4f shows the discharge capacities of Fe,Zr‐NC across a wide range of current densities (2 to 50 mA cm^−2^). Compared with Fe‐NC and Pt/C@RuO_2_, Fe,Zr‐NC consistently exhibited higher discharge voltages under all tested conditions. Even at a high current density of 50 mA cm^−2^, the discharge voltage remained stable and fully recovered upon returning to 2 mA cm^−2^, indicating excellent rate capability. This superior performance is likely attributable to the efficient transport of reactive species and the interconnected porous structure of the Fe,Zr‐NC electrode. The long‐term cycling stability of Fe,Zr‐NC was further evaluated by constant‐current charge‐discharge testing (Figure 4g). The catalyst maintained highly stable performance for 453 h at a current density of 10 mA cm^−2^, significantly outperforming the commercial Pt/C@RuO_2_ counterpart. Additionally, a comparison of ZABs performance with other reported ORR catalysts is summarized in Table S9 (Supporting Information), where Fe,Zr‐NC outperforms most state‐of‐the‐art catalysts. Collectively, these results confirm that Fe,Zr‐NC is a highly durable and efficient bifunctional catalyst with great potential for application in next‐generation zinc‐air batteries.

### 2.5 Degradation Mechanism

**Figure 5:**
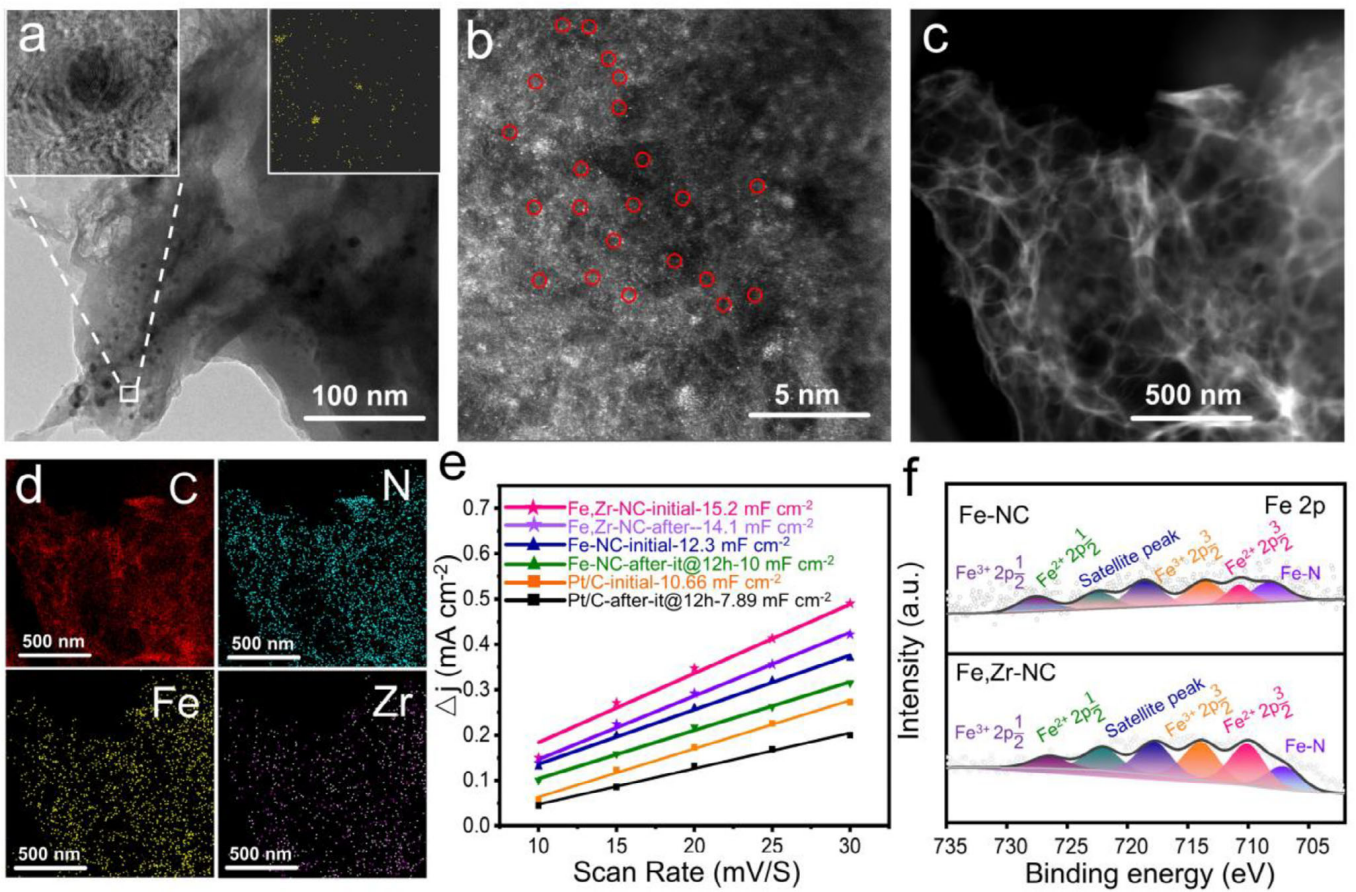
a) TEM images of Fe‐NC (Enlarged detailed enlargement of the particles and EDS of iron, respectively) after the ZABs battery stability test. b) HAADF‐STEM image of Fe,Zr‐NC after 11600 CV cycles. c)TEM image of Fe,Zr‐NC after 11 600 CV cycles, d) EDS mapping images. e) Comparison of CDL before and after i‐t at 12h, f) Fe*2p* spectra after stability.

To investigate the deactivation mechanism of the prepared catalyst, constant‐current charge‐discharge cycling tests were conducted in ZABs. First, the microstructural morphology of the catalysts after long‐term durability testing was characterized. As shown in **Figures** **5**a and S26 (Supporting Information), TEM images of Fe‐NC after 170 h of operation reveal the formation of noticeable metal particles. Aggregation of iron species was further confirmed by the TEM‐EDS mapping (Figure S27, Supporting Information). HRTEM images exhibited graphitic carbon lattice fringes, suggesting that these particles likely resulted from the agglomeration of single iron atoms originally dispersed on the carbon framework. In contrast, no discernible particles or clusters were observed in the TEM images of Fe,Zr‐NC after testing (Figure S28, Supporting Information). EDS elemental mapping (Figure S29, Supporting Information) revealed a homogeneous distribution of Fe and Zr, with no signs of significant aggregation. These findings suggest that the introduction of Zr atoms effectively suppresses the migration and aggregation of Fe atoms from their initial atomic dispersion state.

To gain further insight into the structural evolution of the catalyst after extended cycling, AC HAADF‐STEM was performed on the Fe,Zr‐NC catalyst after 11,600 CV cycles (Figure 5b). The results confirm that atomic dispersion of metal species was maintained, even after prolonged electrochemical operation. As shown in the TEM image (Figure 5c), no nanoparticles were observed, corroborating the absence of metal clustering. EDS mapping (Figure 5d) further demonstrated the uniform distribution of Fe and Zr, reinforcing the hypothesis that Zr plays a critical role in stabilizing Fe active sites and preventing their aggregation. The electrochemically active surface area (ECSA) was estimated via double‐layer capacitance (C*_dl_*) measurements derived from cyclic voltammetry at scan rates ranging from 10 to 30 mV s^−1^ (Figures S30 and S31, Supporting Information). As shown in Figure 5e, the C*_dl_* of Fe,Zr‐NC decreased slightly from 15.2 to 14.1 mF cm^−2^ after durability testing, reflecting a minimal decline of only 1.1 mF cm^−2^. In contrast, Fe‐NC and Pt/C exhibited greater reductions of 2.3 and 2.8 mF cm^−2^, respectively. These results indicate superior structural and electrochemical stability of the Fe,Zr‐NC catalyst, likely attributable to the synergistic interaction between Fe and Zr, which mitigates the dissolution of Fe active sites. To further probe the chemical evolution of the catalysts post‐stability testing, Fe,Zr‐NC and Fe‐NC samples subjected to 11 600 and 5,500 CV cycles, respectively, were analyzed via X‐ray photoelectron spectroscopy (XPS). The survey spectrum (Figure S32, Supporting Information) confirmed the presence of Fe, Zr, N, C, and O in both samples. The C 1*s* spectra (Figure S33, Supporting Information) reveal four deconvoluted peaks, while the N 1*s* spectra are presented in Figure S34. As summarized in Table S10 (Supporting Information), the pyridinic nitrogen content in Fe‐NC dropps significantly decreases to 16.6% after cycling, whereas Fe,Zr‐NC shows only a marginal decrease of 1.4%, indicating greater retention of active nitrogen species in the latter.

High‐resolution Fe 2*p* spectra (Figure 5f) show that Fe‐NC experienced a notable attenuation in the Fe 2*p* satellite peak intensity after durability testing. This reduction is attributed to the loss or transformation of Fe‐N_4_ active sites and the concurrent aggregation of Fe atoms, consistent with TEM observations (Figure 5a). In contrast, Fe,Zr‐NC exhibits a shift of the Fe 2*p* peak toward lower binding energies, indicative of partial reduction of Fe^3^⁺ to Fe^2^⁺. Additionally, the Zr 3*d* spectrum of Fe,Zr‐NC after long‐term cycling (Figure S35, Supporting Information) shows a decrease in intensity, suggesting a partial reduction of Zr^4+^ to Zr^3+^, likely due to electron gain in the reductive ORR environment. Together, these findings confirm that Fe,Zr‐NC maintains excellent chemical and structural integrity under prolonged electrochemical operation. The incorporation of Zr not only stabilizes Fe‐N_4_ active sites but also inhibits Fe dissolution, aggregation, and structural degradation, addressing one of the major limitations of conventional Fe‐based ORR catalysts.

### 2.6 DFT Calculation

**Figure 6:**
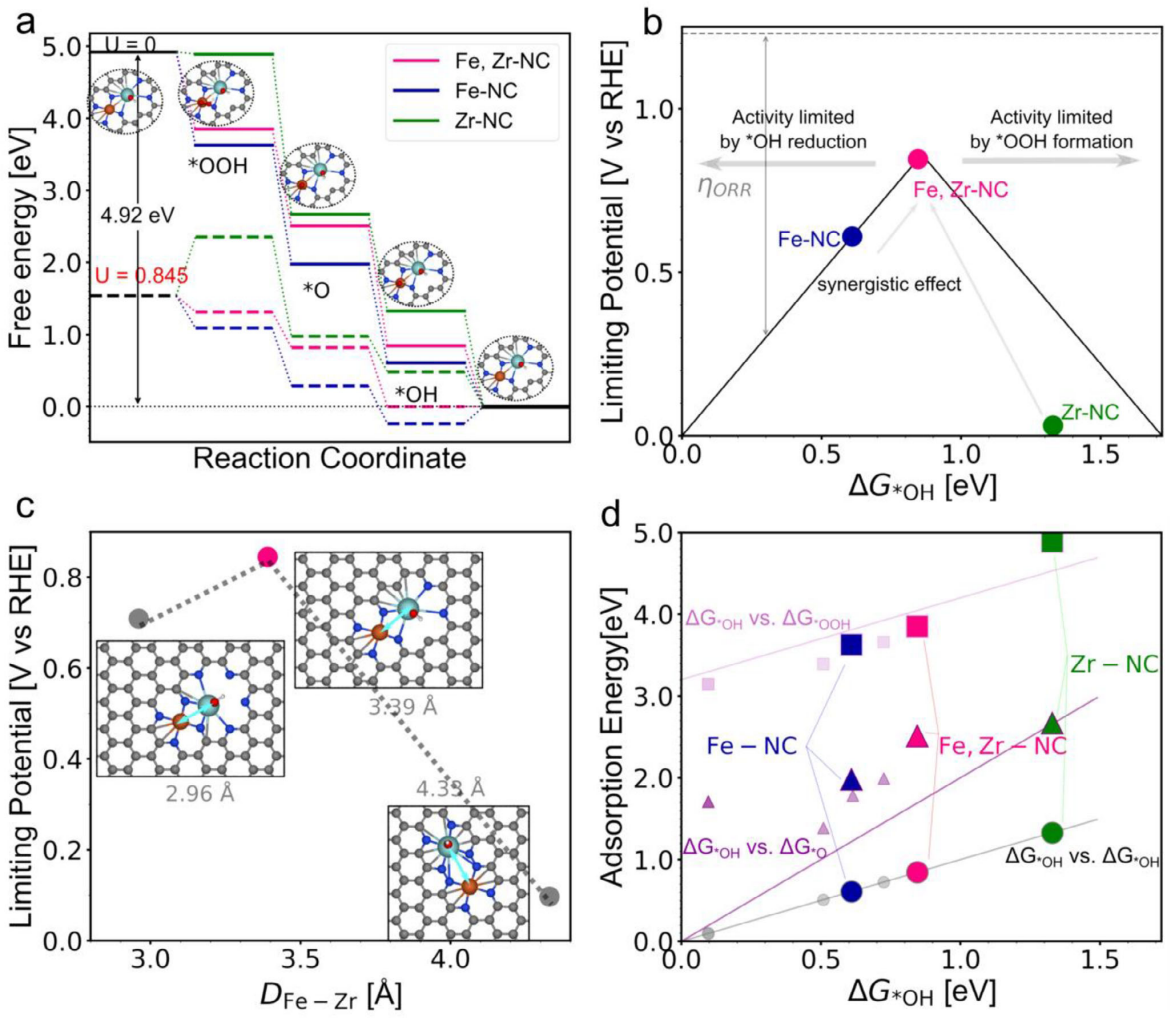
Theoretical insights into the enhanced ORR activity of the Fe,Zr‐NC catalyst. a) Calculated free energy diagrams for the four‐electron ORR pathway on Fe‐NC, Zr‐NC, and Fe,Zr‐NC at U = 0 and U = 0.845 V, indicating the lowest overpotential for Fe,Zr‐NC. b) Volcano plot correlating ORR limiting potential with ^*^OH adsorption free energy (ΔG^*^OH), showing Fe,Zr‐NC positioned near the apex, corresponding to optimal intermediate binding strength. c) Theoretical activity as a function of Fe–Zr interatomic distance, with maximum activity achieved at ≈3.39 Å; activity declines at both shorter (2.96 Å) and longer (4.33 Å) distances. 35d) Adsorption energies of key ORR intermediates (^*^OOH, ^*^O, ^*^OH) on Fe‐NC and Fe,Zr‐NC, demonstrating weaker binding in the dual‐metal system. The solid lines are scaling relations from metal catalysts: gray: ΔG^*^OH vs. ΔG^*^OH; purple: ΔG^*^OH vs. ΔG^*^O (ΔG^*^O = 2^*^ ΔG^*^OH); Plum: ΔG^*^OH vs ΔG^*^OOH (ΔG^*^OOH = ΔG^*^OH + 3.2 eV). The other scatter points (solid filled) are the other Fe,Zr‐NC motifs (configurations see Figure S37, Supporting Information), and they are here just for demonstration.

To elucidate the origin of the enhanced ORR activity in the Fe,Zr‐NC catalyst, DFT calculations were carried out on Fe‐NC, Zr‐NC, and Fe,Zr‐NC model structures. In the Fe,Zr‐NC model, Fe and Zr atoms are co‐coordinated with nitrogen atoms in close proximity within a graphene matrix. The calculated free energy diagrams for the four‐electron ORR pathway (**Figure** **6**a) reveal that the desorption of ^*^OH is the potential‐determining step (PDS) for both Fe‐NC and Fe,Zr‐NC. Notably, Fe,Zr‐NC exhibits a substantially reduced ^*^OH binding energy, resulting in a theoretical limiting potential of 0.845 V, which is significantly higher than that of Fe‐NC (0.729 V) and Zr‐NC (0.518 V). This is further corroborated by the volcano plot (Figure 6b), which correlates the limiting potential with OH adsorption free energy (ΔG^*^OH); Fe,Zr‐NC lies near the apex, suggesting optimized intermediate binding.

To further probe the Fe–Zr synergy, we investigated the influence of interatomic distance on ORR activity. As shown in Figure 6c, the theoretical activity follows a volcano‐type dependence on the Fe–Zr separation, with the highest limiting potential achieved at ≈3.39 Å. This optimal distance closely matches the experimentally determined Zr–Fe path length of 3.30 ± 0.01 Å from EXAFS analysis, confirming the structural validity of the model. In addition, the adsorption energies of other key ORR intermediates (^*^OOH and ^*^O) are consistently weakened on Fe,Zr‐NC compared to Fe‐NC (Figure 6d), which facilitates faster reaction kinetics. The presence of Zr also acts as an auxiliary ^*^OH adsorption site, alleviating ^*^OH poisoning at the Fe center and further improving the reaction turnover. Moreover, projected density of states (PDOS) calculations were performed to probe the electronic origin of the enhanced activity. The extracted Fe d‐band center in Fe,Zr‐NC (−2.65 eV) is downshifted by ≈0.3 eV relative to Fe‐NC (−2.08 eV, Figure S36, Supporting Information). This downshift correlates with weaker ^*^OH adsorption, consistent with the free energy results, and provides a mechanistic explanation for the improved ORR performance. Collectively, these theoretical results suggest that the Fe–Zr dual‐metal synergy modulates the electronic environment of Fe, optimizes intermediate adsorption energies, and enhances both ORR activity and site stability. This mechanistic insight is consistent with the superior electrochemical performance and durability observed in experiments.

## 3 Conclusion

In this study, a Fe–Zr dual‐atom carbon‐based catalyst (Fe,Zr‐NC) was successfully synthesized via a one‐step solid‐state heat treatment, yielding a hierarchically porous carbon framework with atomically dispersed active centers. The catalyst demonstrated outstanding ORR activity in alkaline media, with a half‐wave potential (0.891 V vs. RHE) and a kinetic current density of 48.79 mA cm^−2^—exceeding both Fe‐NC and commercial Pt/C benchmarks. XAS and XPS analyses confirmed that the incorporation of Zr effectively regulates the coordination environment of Fe active sites, stabilizing the active sites and suppressing degradation. DFT calculations further uncover a possible strong synergistic interaction between Fe and Zr: optimal Fe–Zr spacing (≈3.39 Å) and electron donation from Zr effectively tailor the adsorption energies of oxygenated intermediates, positioning Fe,Zr‐NC near the peak of the ORR volcano plot. This electronic modulation not only improves catalytic activity but also mitigates Fe demetallation and carbon corrosion, leading to superior durability. When applied in zinc‐air batteries, Fe,Zr‐NC delivered a high peak power density of 185.7 mW cm^−2^, exceptional cycling stability over 453 h, and excellent rate capability, underscoring its practical viability for energy storage applications. Stability assessments further confirmed that Zr doping effectively suppresses Fe atom aggregation and mitigates nitrogen species loss. Overall, this work provides a promising strategy for the rational design of Fe‐based dual‐atom electrocatalysts and presents a facile approach to fabricating high‐performance, non‐precious metal catalysts for next‐generation energy conversion devices.

## 4 Experimental Section

### Chemicals and Materials Synthesis—*Chemicals*

Heme Chloride was bought from Macklin, 2‐Ethylimidazole, ZrCl_4_ (>95%), Nafion solution (5 wt.%), Zn(NO_3_)_2_.6H_2_O, ethanol, and KOH (99.98%) were purchased from Aladdin, and 20 wt.% Pt/C was bought from Sinero. All chemicals were used directly and without further purification. Ultrapure water (18 MΩ) used in the experiments was supplied by a Millipore System (Millipore Q).

### Chemicals and Materials Synthesis—*Synthesis of Catalyst*

The catalyst synthesis method is as follows: 10 mm 2‐Ethylimidazole, 10.4 mm Zn(NO_3_)_2_·6H_2_O, 0.077 mm heme chloride, 0.051 mm ZrCl_4_ were dissolved in 15 mL of ethanol, then the sample was thoroughly mixed and stirred for 4 h to achieve complete drying. Subsequently, it was transferred to an oven and maintained at 170 °C for 1 h. After cooling to room temperature, the dried sample was collected and designated as the catalyst precursor. The precursor was then subjected to pyrolysis in a tube furnace under an argon atmosphere. The pyrolysis process was conducted at a carbonization temperature of 950 °C, with a heating rate of 5 °C min^−1^, and a dwell time of 2 h. Upon cooling to room temperature, the resulting material was collected and identified as the atomically dispersed dual single‐atom nitrogen‐doped carbon catalyst. Fe‐NC (without ZrCl_4_), Fe‐NC (without C_34_H_32_ClFeN_4_O_4_), and NC (without additional added metals) were prepared in the same way as controls.

### Physical Characterization

Scanning electron microscopy (SEM) was conducted using a Hitachi SU‐70 field‐emission microscope at 5 kV. Transmission electron microscopy (TEM) and energy‐dispersive X‐ray spectroscopy (EDS) mapping were performed on a JEOL F‐200 microscope at 200 kV. High‐angle annular dark‐field scanning transmission electron microscopy (HAADF‐STEM) images were obtained with a Titan Cubed Themis G2 300. Water contact angles were measured using a Dataphysics OCA 20 goniometer. X‐ray diffraction (XRD) patterns were recorded on a Bruker D8 Advance diffractometer (Cu Kα, λ = 1.54178 Å, 40 kV, 200 mA) with a step size of 0.01° over 10–80°. Raman spectra were collected using a RENISHAW inVia spectrometer with 633 nm laser excitation. Nitrogen adsorption–desorption isotherms were measured at 77 K using a Micromeritics ASAP 2020. Specific surface area and pore structure were analyzed by the BET and BJH methods, respectively. X‐ray photoelectron spectroscopy (XPS) was carried out on a Microlab 350 system using a Mg Kα source. Metal contents were quantified by inductively coupled plasma optical emission spectrometry (ICP‐OES).

### Electrochemical Measurements

Electrochemical measurements were performed by using an electrochemical workstation (a Bio‐Logic VMP‐300) and electrocatalysis equipment (PINE) in 0.1 m KOH solution with a three‐electrode system. The working electrode was an RRDE coated with a catalyst film. A Hg/HgO and a carbon rod were used, respectively, as the reference electrode and the counter electrode. The following equation was used to calculate the reference hydrogen electrode (RHE): E_RHE_ = E*_Hg/HgO_* +0.098+0.0592*pH*.

The catalyst ink was prepared by mixing 100 uL Milli Q water, 140 uL ethanol, 10 uL 5 wt.% Nafion solution and 1 mg catalysts, followed by ultrasonication for 2 h. Then, 12 µL of the ink was uniformly loaded onto the Al_2_O_3_ polished glassy‐carbon electrode (diameter = 0.55 cm), which was used as the working electrode with a loading mass of 0.102 mg cm^−2^. The same formula was utilised for the 20 wt.% Pt/C control sample. The specific methods of electrochemical testing were referred to previous published work^[^ ^29^ ^]^ (see details in Supporting Information).

### Zinc–Air Battery Measurements

Zinc–air batteries were assembled with zinc foil as the anode and Fe,Zr–NC as the air cathode. The catalyst ink (1 mg catalyst, 240 µL ethanol, 10 µL 5 wt.% Nafion) was drop‐cast onto a 1 cm^2^ GDL and dried for 12 h (loading: 1 mg cm^−2^). The battery used 6 m KOH as the electrolyte. A Pt/C–RuO_2_ (1:1) cathode was used as a control. Polarization curves were measured by LSV (5 mV s^−1^), and cycling tests were conducted at 10 mA cm^−2^ with 10 min discharge/charge intervals. Two batteries in series powered a 3.5 V LED under ambient air.

### Density Functional Theory (DFT) Calculations

The computational analysis was carried out using the grid‐based projector‐augmented wave (GPAW) method, a DFT code based on a projected augmented wave (all‐electron frozen core approximation) method integrated with the atomic simulation environment (ASE).^[^ ^30^ ^]^ The Bayesian error estimation functional with van der Waals correlation (BEEF–vdW) was used as an exchange‐correlation functional.^[^ ^31^ ^]^ The wave functions were represented on a uniform real‐spaced grid with 0.18 Å grid spacing under a (3 × 3 × 1) k‐point sampling. The electronic spins are treated separately, and a vacuum of at least 10 Å was employed. The quasi‐Newton minimization scheme with spin included was employed for the geometry optimizations, and the systems were relaxed until the forces were less than 0.05 eV Å^−1^.

## Conflict of Interest

The authors declare no conflict of interest.

## Supporting information



Supporting Information

## Data Availability

The data that support the findings of this study are available from the corresponding author upon reasonable request.
