# Supplementary material for: Atomically Dispersed Zr‐N Moieties Modulate Fe Coordination for Robust Oxygen Reduction Electrocatalysis
Source: Adv Sci (Weinh). 2025 Sep 29;12(47):e12381. doi: 10.1002/advs.202512381 (PMC12713020; doi:10.1002/advs.202512381)
Supplement: Supplementary file 1 — Supporting Information [file ADVS-12-e12381-s001.docx]

**Atomically Dispersed Zr–N Moieties Modulate Fe Coordination for Robust Oxygen Reduction Electrocatalysis**

*Siqi Qiu ^a1^, Hao Wan^b1^, Yuechao Yao^a*^, Xiao Xu ^a^, Zhangjian Li ^a^, Yongbiao Mu^c^, Biaolin Peng^d^, Hongliang Wu^a^, Jizhao Zou^a*^, Lin Zeng^c*^*

^a^*Guangdong Provincial Key Laboratory of New Energy Materials Service Safety & Shenzhen Key Laboratory of Special Functional Materials & Shenzhen Engineering Laboratory for Advance Technology of Ceramics, College of Materials Science and Engineering, Shenzhen University, Shenzhen, Guangdong 518060. P.R. China.*

*^b^Fritz-haber-institut der max-planck-gesellschaft, German*

*^c^ Department of Mechanical and Energy Engineering, Southern University of Science and Technology, Shenzhen, Guangdong 518060. P.R. China.*

*^d^School of Advanced Materials and Nanotechnology, Xidian University, Xi'an, 710126, China*

Corresponding author: (Yuechao Yao) yaoyuechao@szu.edu.cn, (Jizhao Zou) zoujizhao@szu.edu.cn, (Lin Zeng) zengl3@sustech.edu.cn.

**1.Experimental Section**

**XAFS measurements**

The X-ray absorption fine structure (XAFS) of Fe and K-edge were performed on BL13SSW/BL11B station in Shanghai Synchrotron Radiation Facility and in the Beijing Synchrotron Radiation Facility. Data reduction, data analysis, and EXAFS fitting were performed and analyzed with the Athena and Artemis programs of the Demeter data analysis packages (reference 1：B. Ravel and M. Newville, ATHENA, ARTEMIS, HEPHAESTUS: data analysis for X-ray absorption spectroscopy using IFEFFIT to fit the EXAFS data. The energy calibration of the sample was conducted through standard Fe foil, which as a reference was simultaneously measured. A linear function was subtracted from the pre-edge region, then the edge jump was normalized using Athena software. The χ(k) data were isolated by subtracting a smooth, third-order polynomial approximating the absorption background of an isolated atom. The k2-weighted χ(k) data were Fourier transformed after applying a HanFeng window function (Δk = 1.0). For EXAFS modeling, the global amplitude EXAFS (CN, R, σ^2^ and ΔE_0_) were obtained by nonlinear fitting, with least-squares refinement, of the EXAFS equation to the Fourier-transformed data in R-space, using Artemis software, EXAFS of the Fe foil was fitted and the obtained amplitude reduction factor S02 value (0.705) was set in the EXAFS analysis to determine the coordination numbers (CNs) in sample.

**Electrocatalytic measurements**

Electrochemical measurements were conducted using a standard three-electrode rotating ring-disk electrode (RRDE, geometric area: 0.2376 cm²) system. A glassy carbon electrode served as the working electrode, a Hg/HgO electrode as the reference electrode, and a graphite rod as the counter electrode. The catalyst ink was prepared by dispersing the catalyst powder in a mixture of 100 μL Milli-Q water, 140 μL ethanol, and 10 μL of 5 wt% Nafion solution, followed by sonication for ~2 h. The resulting suspension was drop-cast onto the glassy carbon electrode and dried for ~30 min prior to testing.

For ORR measurements, the working electrode was first preconditioned by cyclic voltammetry (CV) between 0.2 and –0.8 V vs. Hg/HgO at 50 mV s⁻¹ until a stable response was achieved. Electrochemical activation was then performed by 30 CV cycles in N_2_-saturated 0.1 M KOH. The electrolyte was subsequently saturated with O_2_ by bubbling for 30 min, and an O_2_ flow of 20 mL min⁻¹ was maintained during measurements. Linear sweep voltammetry (LSV) was carried out in O_2_-saturated 0.1 M KOH at a scan rate of 5 mV s⁻¹ with a rotation speed of 1600 rpm. Background CV and LSV profiles were recorded in N_2_-saturated electrolyte after 20 min of N_2_ bubbling, and the final ORR polarization curves were obtained by subtracting the N_2_ background from the O_2_ LSV data.

Polarisation curves were plotted as overpotential (η) versus the log current (log[J]) to obtain the Tafel plots. The Tafel slope (b) was obtained by fitting the linear portion of the Tafel plots to the Tafel equation (η=b log[J] + a). Rotation disk electrode (RRDE) measurement was performed at a sweep rate of 10 mV s−1 with different rotating speeds (400–2025 rpm). The electron transfer number (n) and kinetic current density (JK) were determined by the Koutecky-Levich equation.
 $\frac{\text{1}}{\text{J}}\text{=}\frac{\text{1}}{\text{J}_{\text{L}}}\text{+}\frac{\text{1}}{\text{J}_{\text{K}}}\text{=}\frac{\text{1}}{\text{B}\text{ω}^{\frac{\text{1}}{\text{2}}}}\text{+}\frac{\text{1}}{\text{J}_{\text{K}}}$

where J is the measured current density, J_K_ and J_L_ are the kinetic-limiting current density and diffusion-limiting current densities and ω represents the electrode rotation rate.

$$\text{B=0.62nF}\text{C}_{\text{0}}\text{D}_{\text{0}}^{\text{2/3}}\text{V}^{\text{-1/6}}$$

B could be determined from the slope of the Koutecky-Levic. Here, n is the number of transferred electrons, F is the Faraday constant (96485 C mol^−1^ ), C_0_ is the bulk concentration of O_2_ (1.2×10^−6^ mol cm^−3^), D_0_ is the diffusion coefficient of O_2_ (1.93×10^−5^ cm^2^ s ^−1^ ) in 0.1M KOH and ν is the kinematic viscosity of the electrolyte (0.01 cm^−2^ s^−1^ ). For the RRDE test, the disk electrode was scanned negatively (10 mV s^−1^ ), causing the electrooxidation of hydrogen peroxide (H_2_O_2_). Hydrogen peroxide yield and the electron transfer number (n) were calculated based on the following equations (3) and (4), respectively:

$$\text{H}_{\text{2}}\text{O}_{\text{2}}\left( \text{\%} \right)\text{=200\%×}\frac{\frac{\text{I}_{\text{R}}}{\text{N}}}{\text{I}_{\text{D}}\text{+}\frac{\text{I}_{\text{R}}}{\text{N}}}$$

$$\text{n=4×}\frac{\text{I}_{\text{D}}}{\text{I}_{\text{D}}\text{+}\frac{\text{I}_{\text{R}}}{\text{N}}}$$

where I_D_ and I_R_ are the disk and ring currents, and N is the ring collection efficiency of the Pt ring (N = 0.37)^[1]^

The turnover Frequency (TOF) at 0.8 V were calculated from the equations:

$$\text{TOF=}\frac{\text{J}_{\text{K}}\text{×s}}{\text{4×F×N}}$$

$\text{N=W×}\frac{\text{m}}{\text{M}}$ *(for single atom catalysts)*

Where J_k_ is the kinetic current at 0.8 V, s is the disk area of RRDE (0.2376 cm^2^), F is the Faraday constant (96485 C mol^−1^), N is the number of active sites, W is the metal content of catalyst measured by ICP-OES, m is the loading of the catalyst on the electrode, M is relative atomic mass.

Methanol tolerance was tested by introducing 6 mL of methanol into an O₂-saturated 0.1 M KOH electrolyte. We conducted a chronoamperometry test to study the electrochemical properties of this material. In a saturated O₂ atmosphere, a constant voltage of 0.4 V was applied while the rotating disk was maintained at a rotational speed of 250 rpm. Durability tests for Fe, Zr-N-C, Fe-NC and Pt/C were performed by continuous CV scans, ranging from -0.164 V to 1.164 V vs. RHE (O_2_-saturated 0.1 M KOH, room temperature).

**Computation methods**

The computational analysis was carried out using the grid-based projector-augmented wave (GPAW) method, a DFT code based on a projected augmented wave (all-electron frozen core approximation) method integrated with the atomic simulation environment (ASE).^[1]^ The Bayesian error estimation functional with van der Waals correlation (BEEF–vdW) was used as an exchange-correlation functional.^[2]^ The wave functions were represented on a uniform real-spaced grid with 0.18 Å grid-spacing under a (3 ×3 × 1) k-point sampling. The electronic spins are treated separately, and a vacuum of minimum 10 Å was employed. The quasi-Newton minimization scheme with spin included was employed for the geometry optimizations, and the systems were relaxed until the forces were less than 0.05 eV/Å.

The formation energies of the ORR intermediates at 0 K are calculated with respect to H_2_O(g) and H_2_(g) in the following way:

ΔE_*OH_ = E_*OH_ - E_*_ − (E_H2O(g)_ − 1/2E_H2(g)_)

ΔE_*O_ = E_*O_ - E_*_ − (E_H2O(g)_ − E_H2(g)_)

ΔE_*OOH_ = E_*OOH_ - E_*_ − (2E_H2O(g)_ − 3/2E_H2(g)_)

In order to obtain the Gibbs free energies at room temperature we have added zero point energy correction and entropy contributions according to ref 24^[3]^, where H2O(g) is considered at the vapor pressure of H2O(l) at 300 K. Furthermore, we have accounted for water induced stabilization of the OH and OOH intermediates corresponding to 0.3 eV per intermediate.^[4]^ At U = 0 V the Gibbs free energies are given by:

ΔG_*OH_ = ΔE_*OH_ + 0.35 -0.3

ΔG_*O_ = ΔE_*O_ + 0.05

ΔG_*OOH_ = ΔE_*OOH_ + 0.40 -0.3

The analyses of ΔG_*OOH_ are identical, and we therefore introduce a variable that refers to both formation energies, ΔG_*OOH_. Following the CHE,^25^ the Gibbs free energies can obtained for an arbitrary value of the potential according to

ΔG_i_(U) = ΔG_i_ − neU

where n is the number of electrons involved in the electrochemical reaction and e is the electron charge.

**Reagent**

| **Reagent** | **Manufacturer** | **specification** |
| --- | --- | --- |
| Methanol | Aladdin | 99.5% |
| 2-Ethylimidazole | Johnson Matthey | 98% |
| Zinc nitrate hexahydrate (Zn(NO_3_)_2_·6H_2_O) | Aladdin | 99.99% |
| zinc acetate (Zn(OAc)_2_) | Aladdin | 99% |
| heme chloride | Macklin | 98% |
| Zirconium chloride | Aladdin | 99.8% |
| Potassium hydroxide | Aladdin | 99.8% |
| Nafion | Sigma-Aldrich | 5 wt% |

**Characterization**

| **Project** | **Equipment or technology** | | **Equipment model** |
| --- | --- | --- | --- |
| Morphologies and structures | SEM | | Hitachi (SU-70) |
| Morphologies and structures | TEM | | JEOL (JEM-F200) |
| Morphologies and structures | HAADF-STEM | | Titan Cubed Themis (G2 300) |
| Phase structure | XRD | | Bruker (D8 Advance) |
| Chemical signal | XPS | Thermo Fisher (Microlab 350) | |
| Surface area | BET | | Micromeritics (ASAP 2020) |
| Spectral analysis | Raman spectra | | Renishaw (Renishaw InVia Raman) |
| Electrochemical performance | Electrochemical workstation | | Bio-Logic (VMP-300) |
| Electrochemical performance | Battery test system | | Neware (CT-4008) |


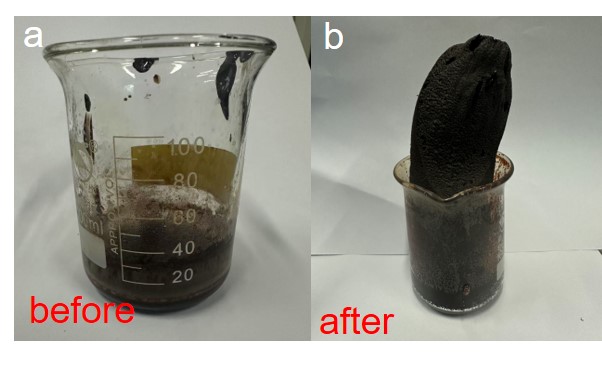


**Figure S1.** The digital photo of heat-treated organic-carbon precursor and before.


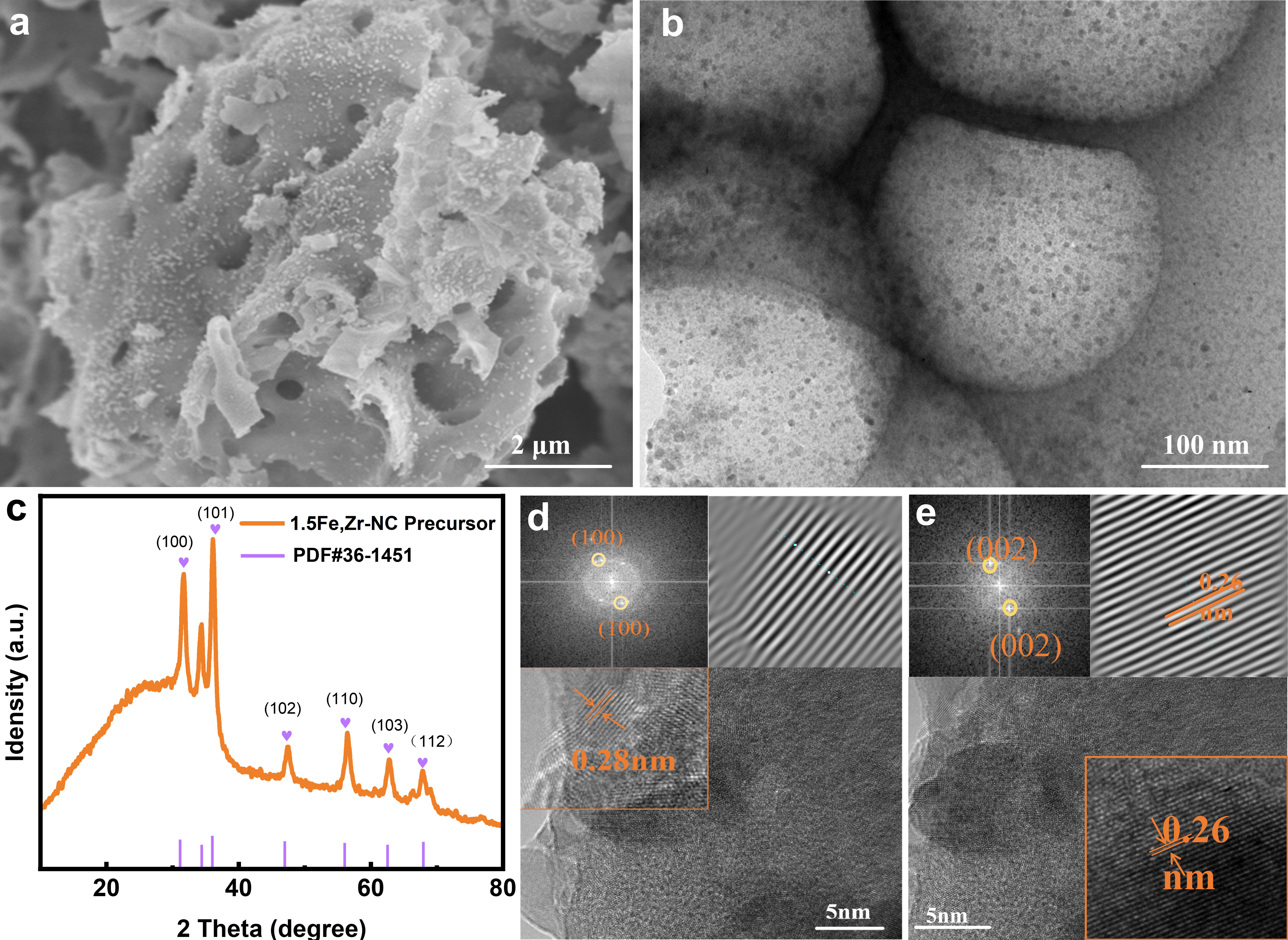


**Figure S2.** a) SEM image of precursor of Fe, Zr-N-C, b) TEM image of precursor of Fe, Zr-N-C, c) XRD patterns of precursor of Fe, Zr-N-C, d-e) Lattice fringe diffraction pattern of a Fe, Zr-N-C precursor.


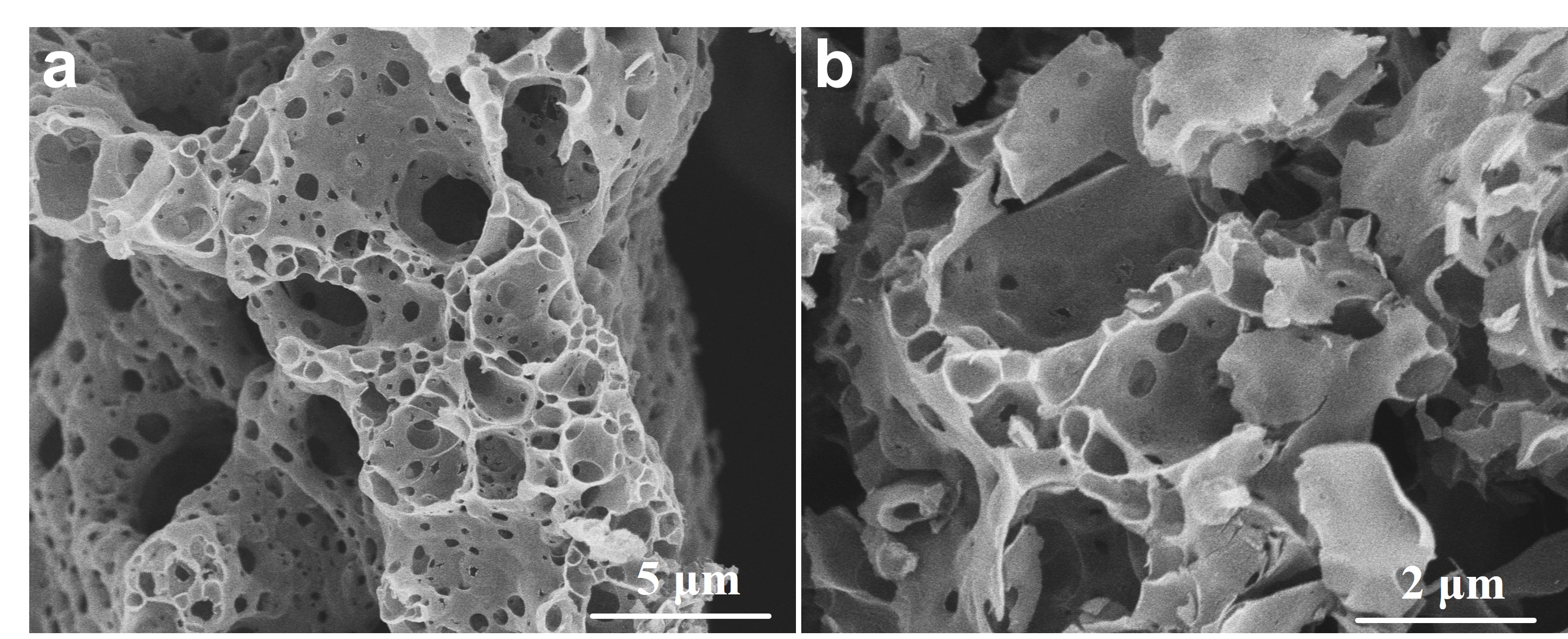


**Figure S3.** SEM image of Fe,Zr-NC


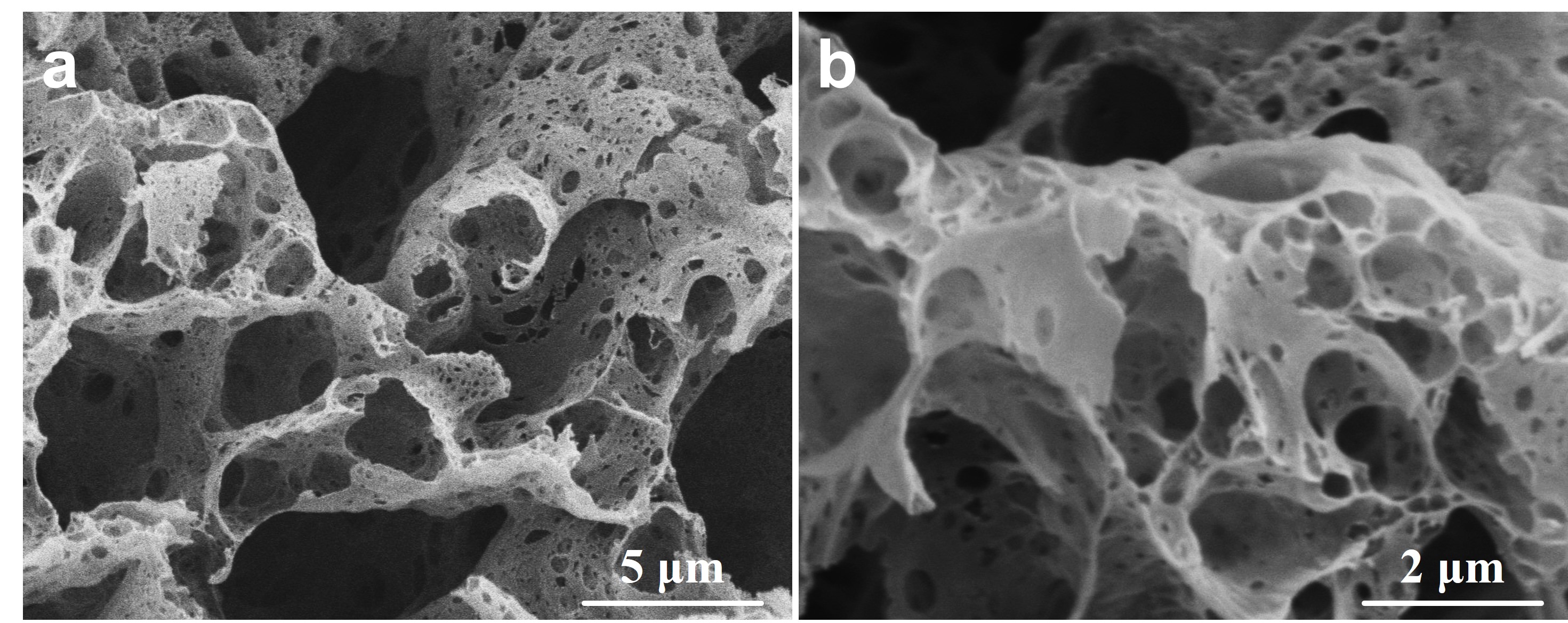


**Figure S4.** SEM image of Fe-N-C


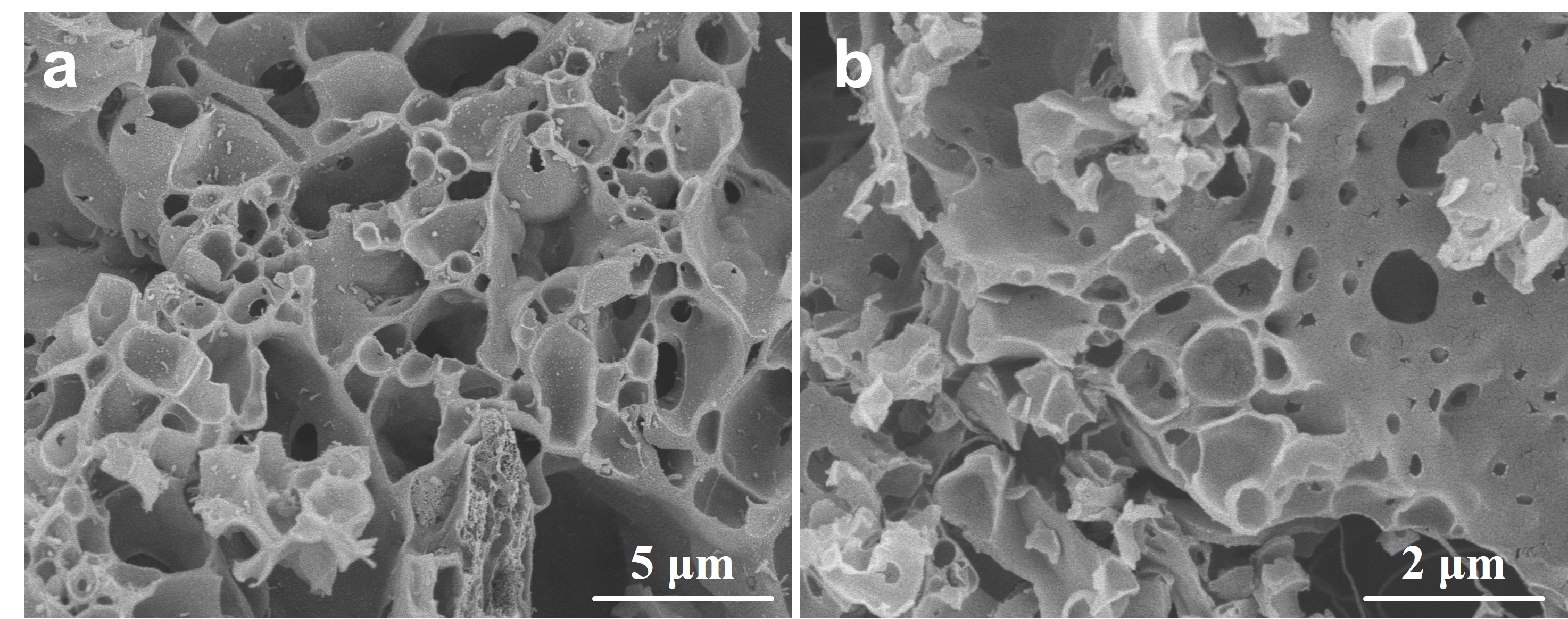


**Figure S5.** SEM image of Zr-N-C


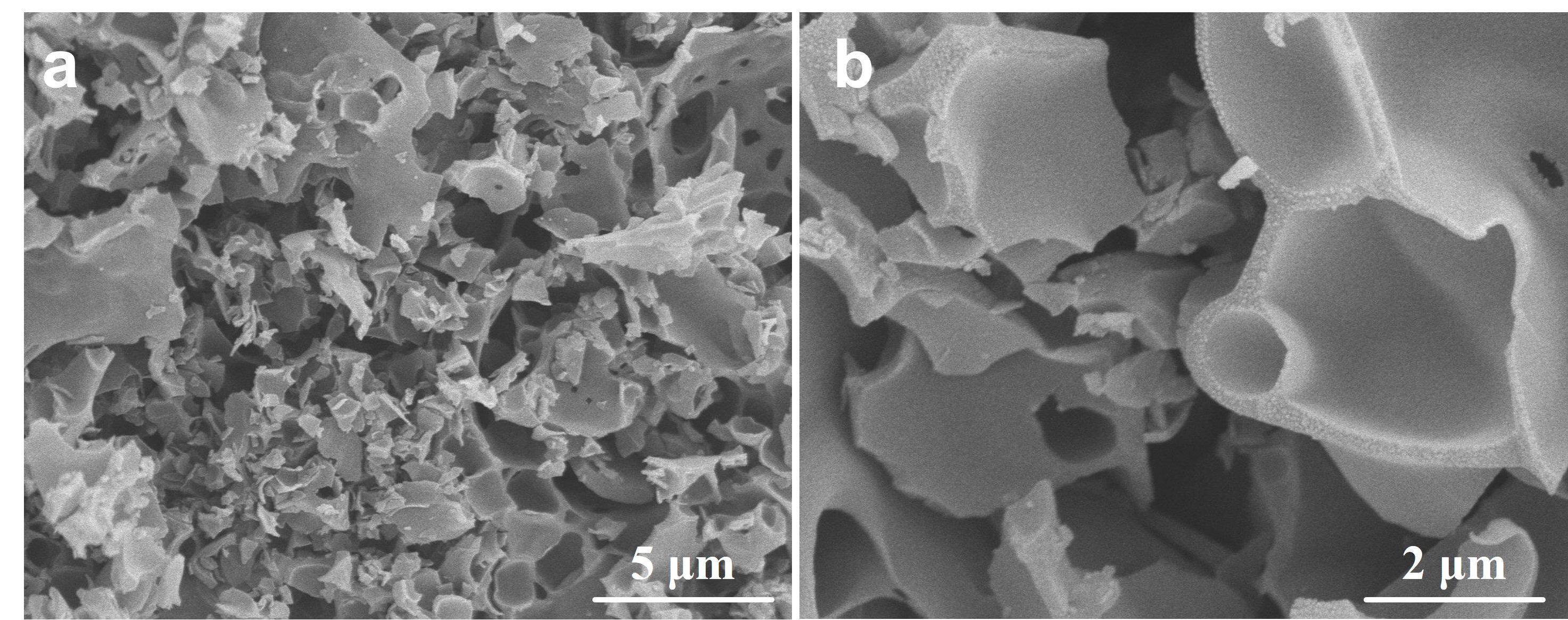


**Figure S6.** SEM image of N-C

**Table S1.** The inductively coupled plasma optical emission spectrometry (ICP-OES) results for different catalysts prepared at 950 ^o^C carbonization temperature.

| **Sample** | **Fe (wt%)** | **Zr (wt%)** |
| --- | --- | --- |
| Fe,Zr-NC | 1.881 | 1.184 |
| Fe -NC | 2.112 | - |
| Zr-NC | - | 1.188 |

**Table S2.** The ICP-OES results for Fe,Zr-NC catalysts prepared at various carbonization temperatures.

| **Sample** | **Zn (wt%)** |
| --- | --- |
| Fe,Zr-NC-900 | 3.554 |
| Fe,Zr-NC-950 | 1.877 |
| Fe,Zr-NC-1000 | 0.032 |


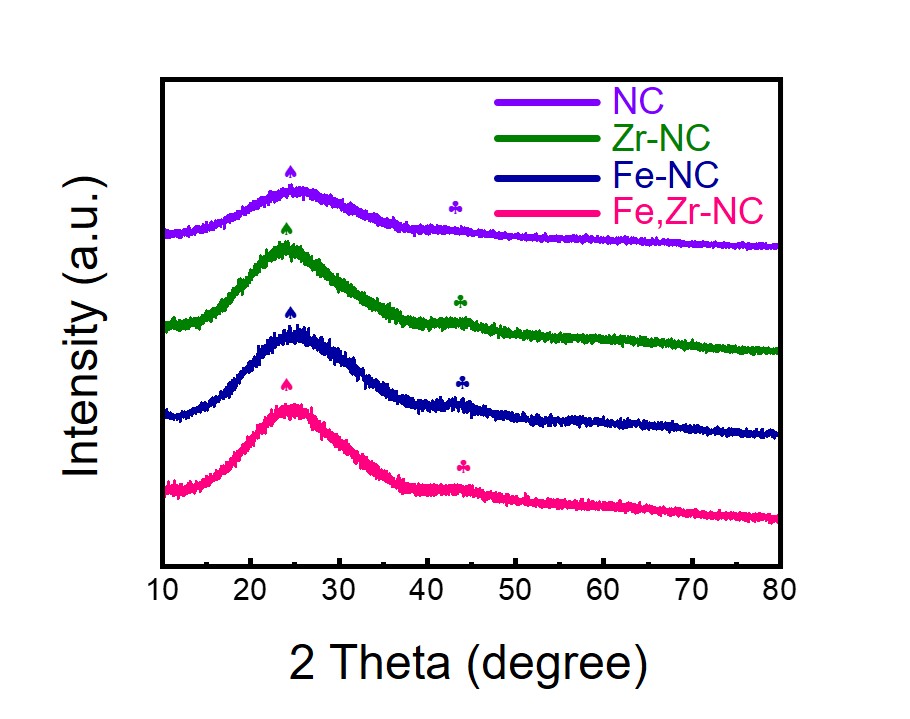


**Figure S7.** XRD pattern for Fe, Zr-NC, Fe-NC, Zr-NC and N-C.

There are only two major diffraction peaks around 24° and 44°, which are attributed to the (002) and (101) planes of graphite. No peaks associated with Fe or Zr species were observed, indicating the absence of Fe- and Zr-related clusters or nanoparticles.


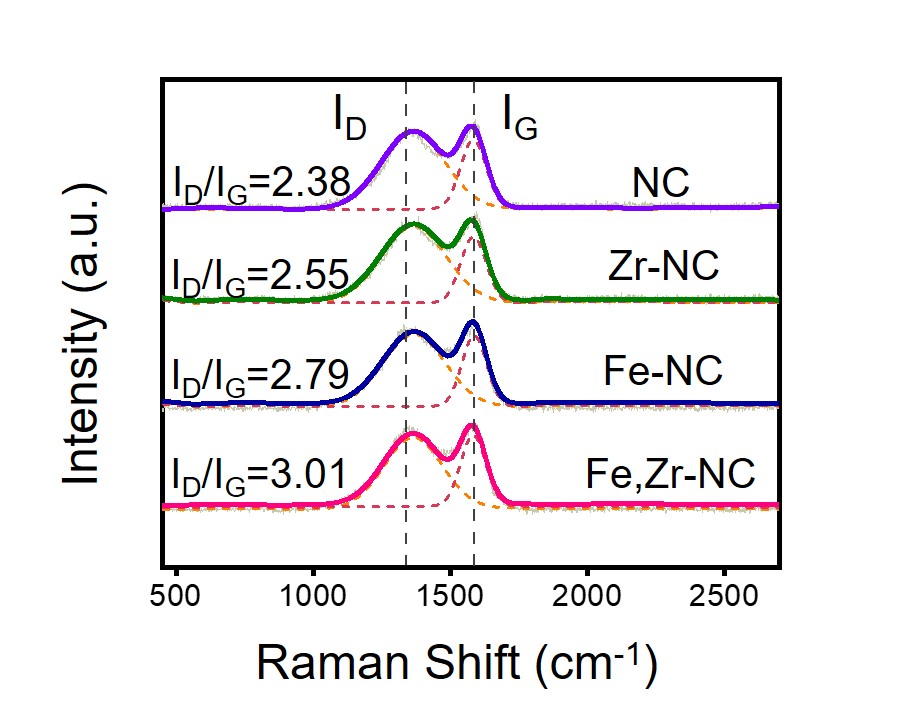


**Figure S8.** Raman spectra for Fe, Zr-NC, Fe-NC, Zr-NC and N-C.

**
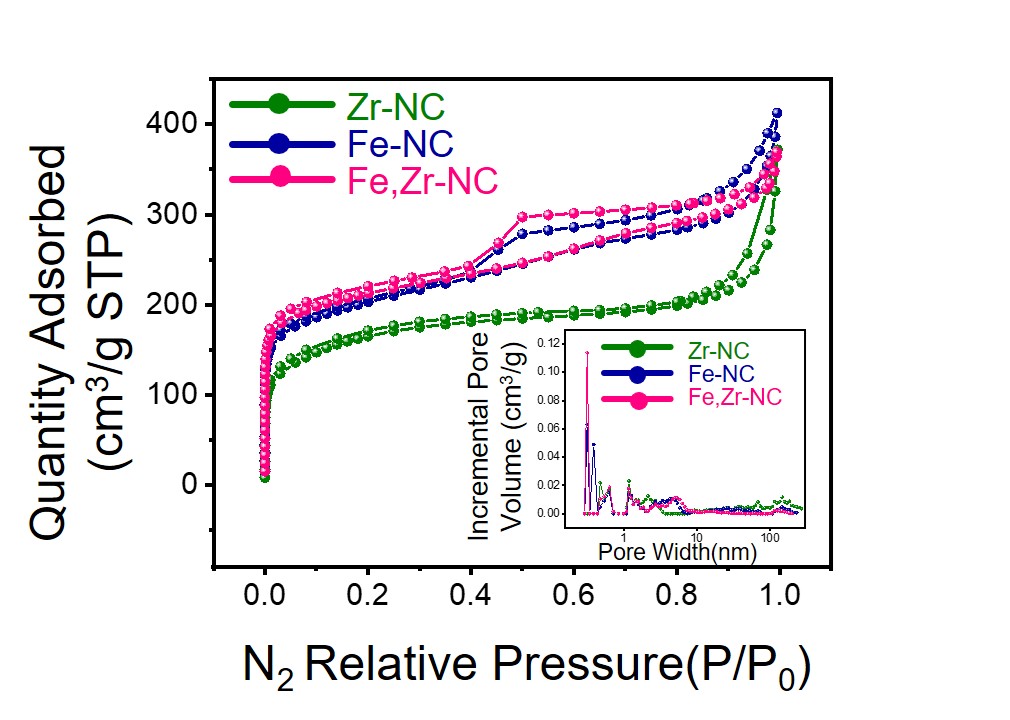
**

**Figure S9.** N_2_ adsorption–desorption isotherm for Fe, Zr-NC, Fe-N-C, Zr-N-C(inset: corresponding pore size distribution)

**Table S3.** BET specific surface area and hole size of the catalysts

| Sample | BET Surface Area (m^2^ g^-1^) | Micropore Area  (m^2^ g^-1^) | Micropore volume  (cm^-3^ g^-1^) | Average pore Size (nm) |
| --- | --- | --- | --- | --- |
| Fe,Zr-NC | 668.82 | 416.82 | 0.209 | 3.159 |
| Fe-NC | 668.39 | 355.67 | 0.177 | 3.57 |
| Zr-NC | 533.39 | 399.05 | 0.19 | 3.68 |


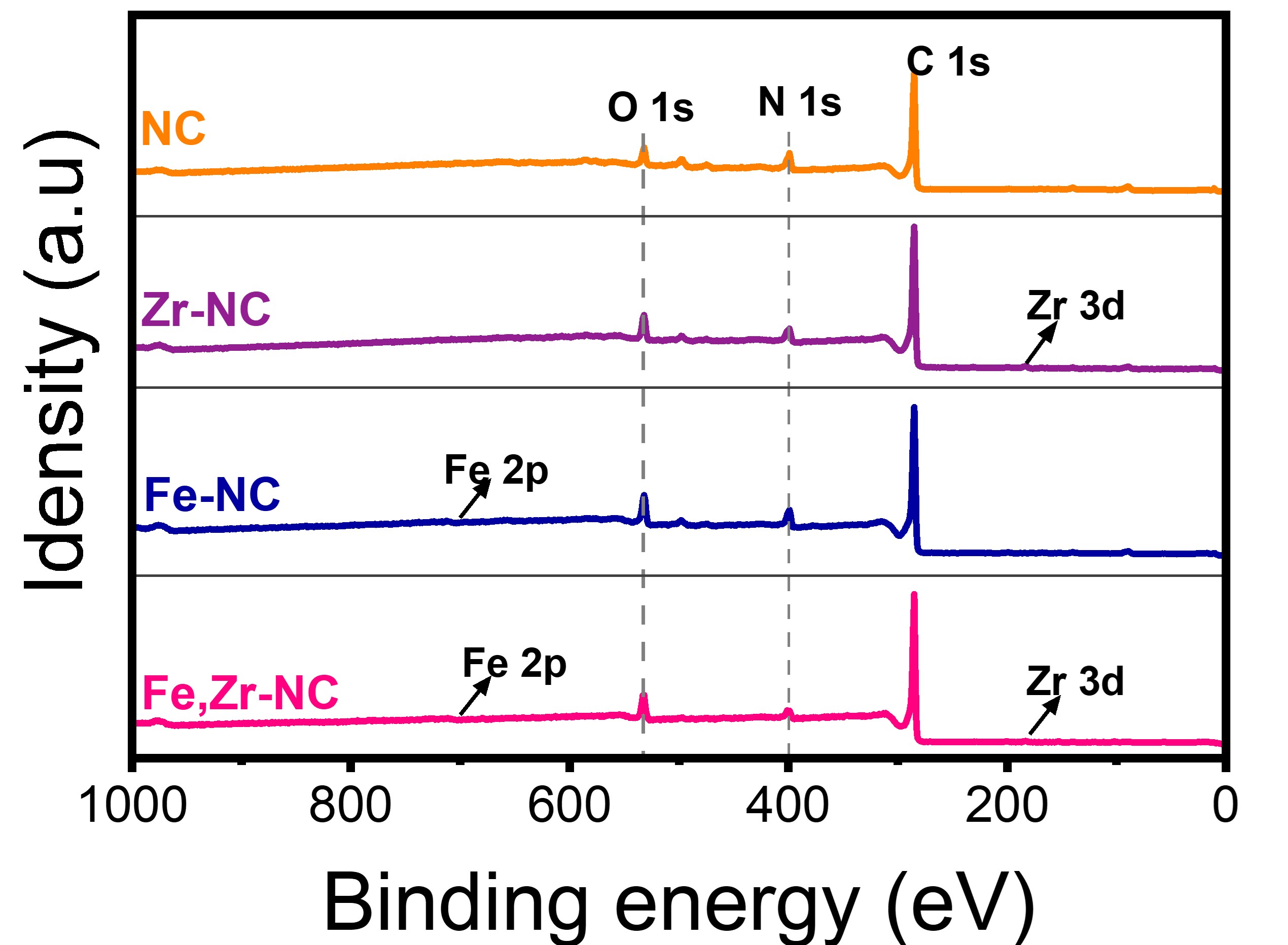


**Figure S10.** XPS survey spectra of NC, Zr-NC, Fe-NC and Fe,Zr-NC


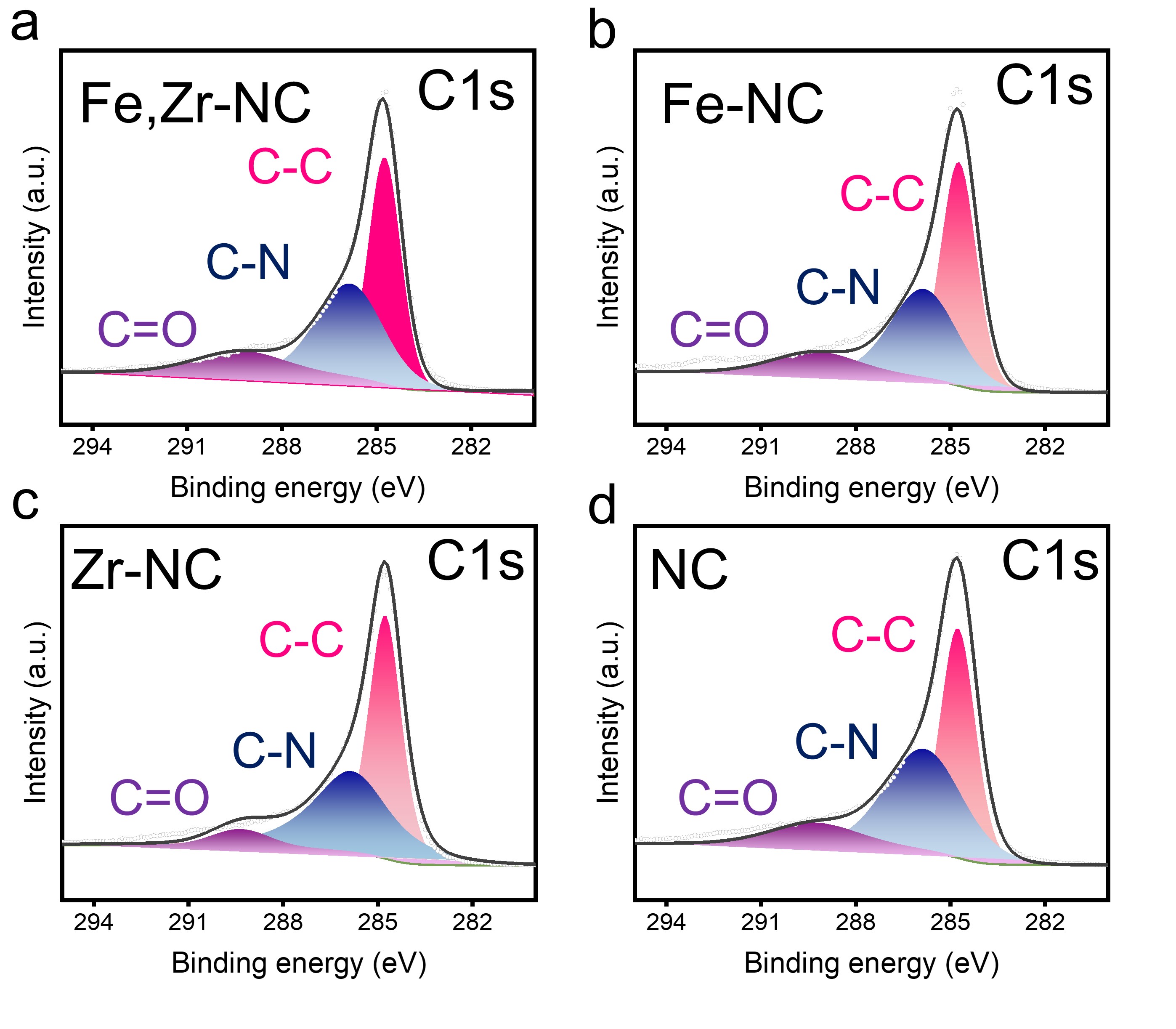


**Figure S11.** High-resolution XPS C 1*s* spectra of a) Fe,Zr-N--C b) Fe –N-C, c) Zr-NC, and d) N-C.

**
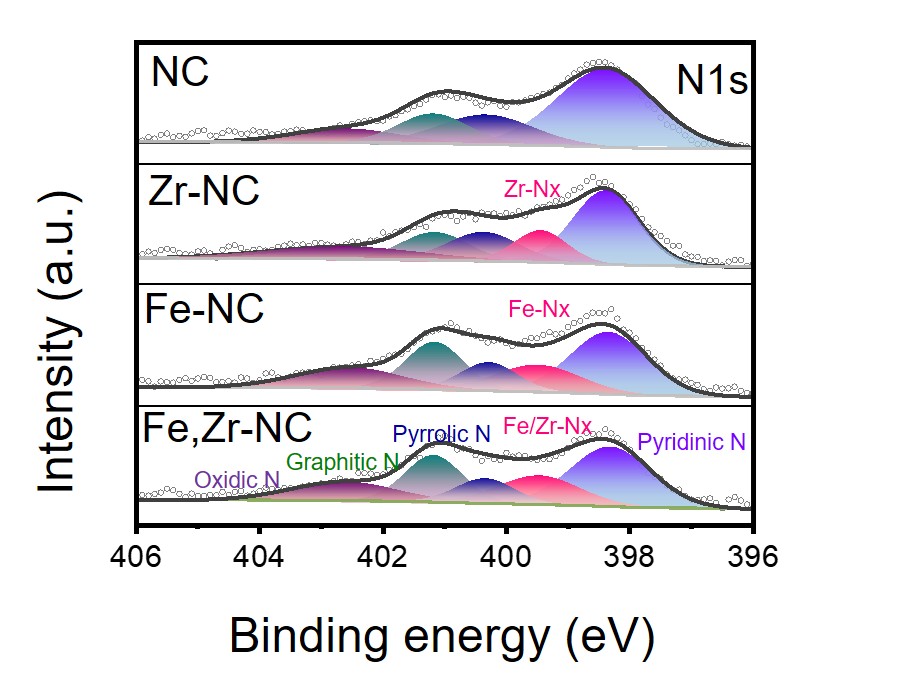
**

**Figure S12.** N 1s XPS spectra of Fe, Zr-N-C, Fe-N-C, Zr-N-C, and N-C.

**Table S4.** The nitrogen content of various catalyst.

| Sample | Oxidized-N | Graphitic-N | Pyrrolic-N | M-N | Pyridinic-N |
| --- | --- | --- | --- | --- | --- |
| Fe, Zr-NC | 13.4 | 19.5 | 13.8 | 12.6 | 40.6 |
| Fe-N-C | 19.1 | 14.1 | 11.3 | 16.4 | 39 |
| Zr-NC | 16.9 | 19.7 | 11.8 | 16.9 | 34.6 |


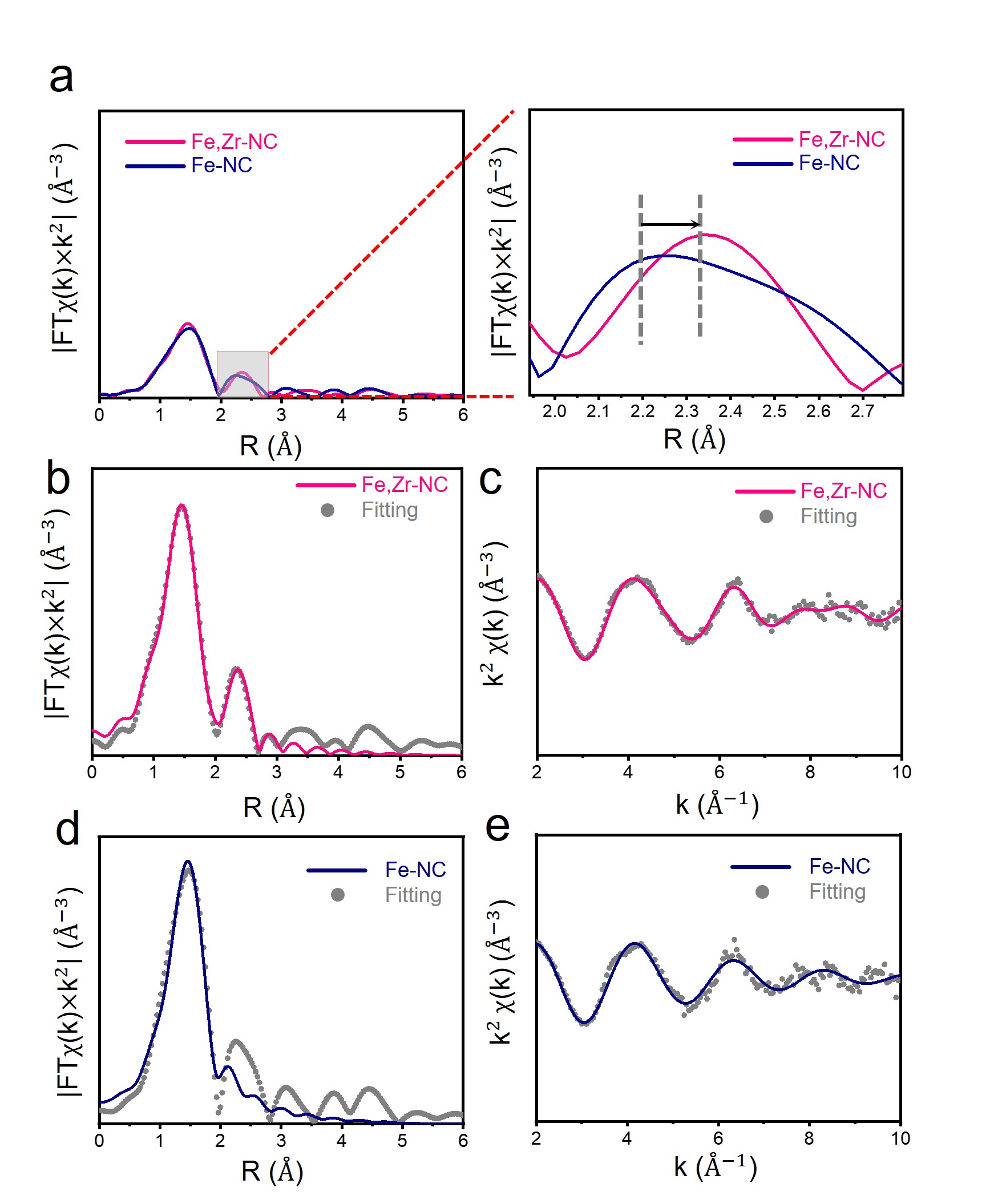


**Figure S13**. a) Rspace local enlargement map of Fe-Zr-NC and Fe-NC; EXAFS spectral fitting for Fe,Zr-NC in (b) q space and (c) k space at Fe K-edge; EXAFS spectra fitting for Fe-NC in (d) q space and (e) k space at Fe K-edge


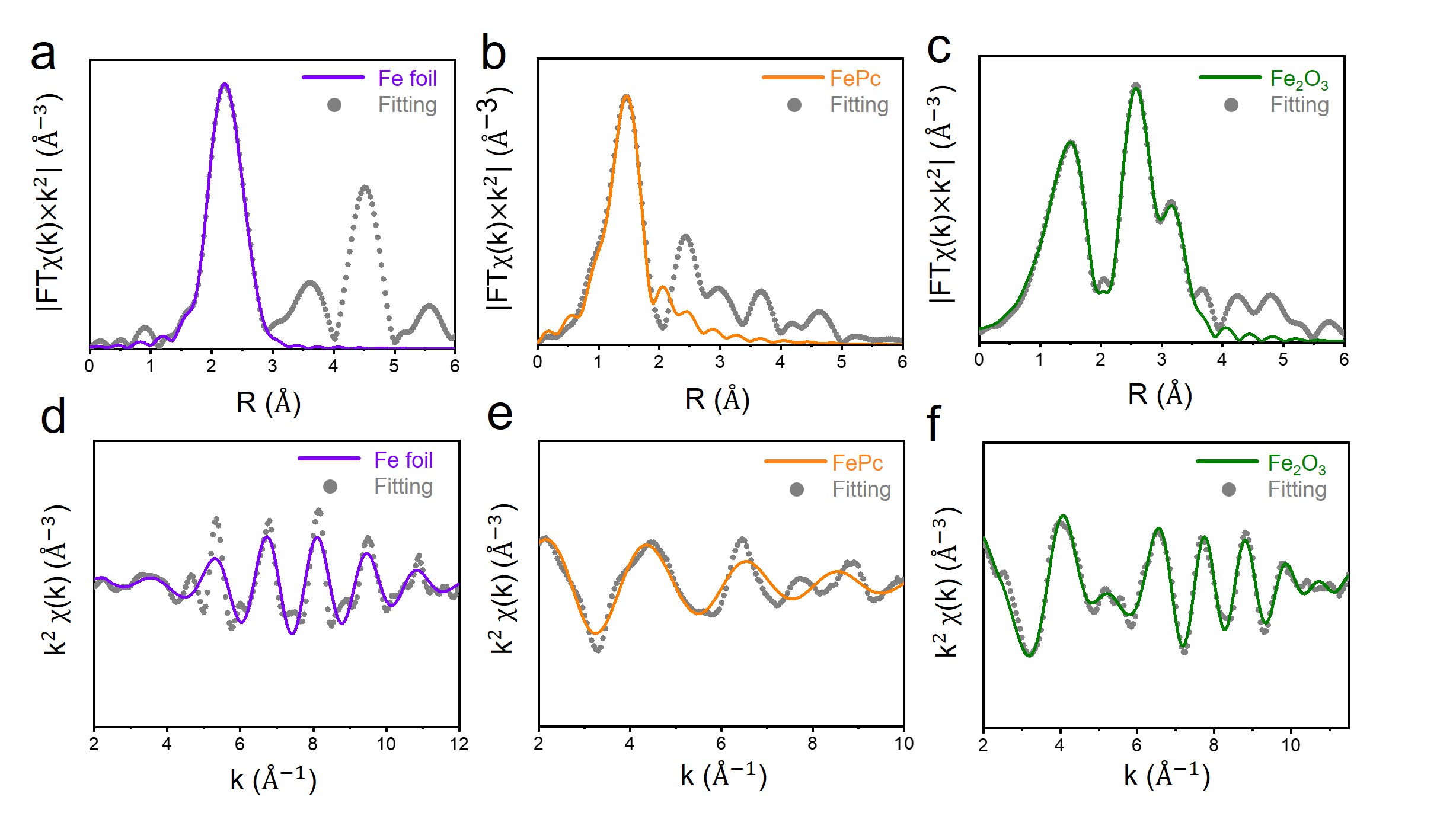


**Figure S14**. EXAFS spectral fitting for Fe foil (a) , FePc (b) and Fe_2_O_3_ (c) in q space at Fe K-edge; EXAFS spectra fitting for Fe foil (d) , FePc (e) and Fe_2_O_3_ (f) in k space at Fe K-edge.


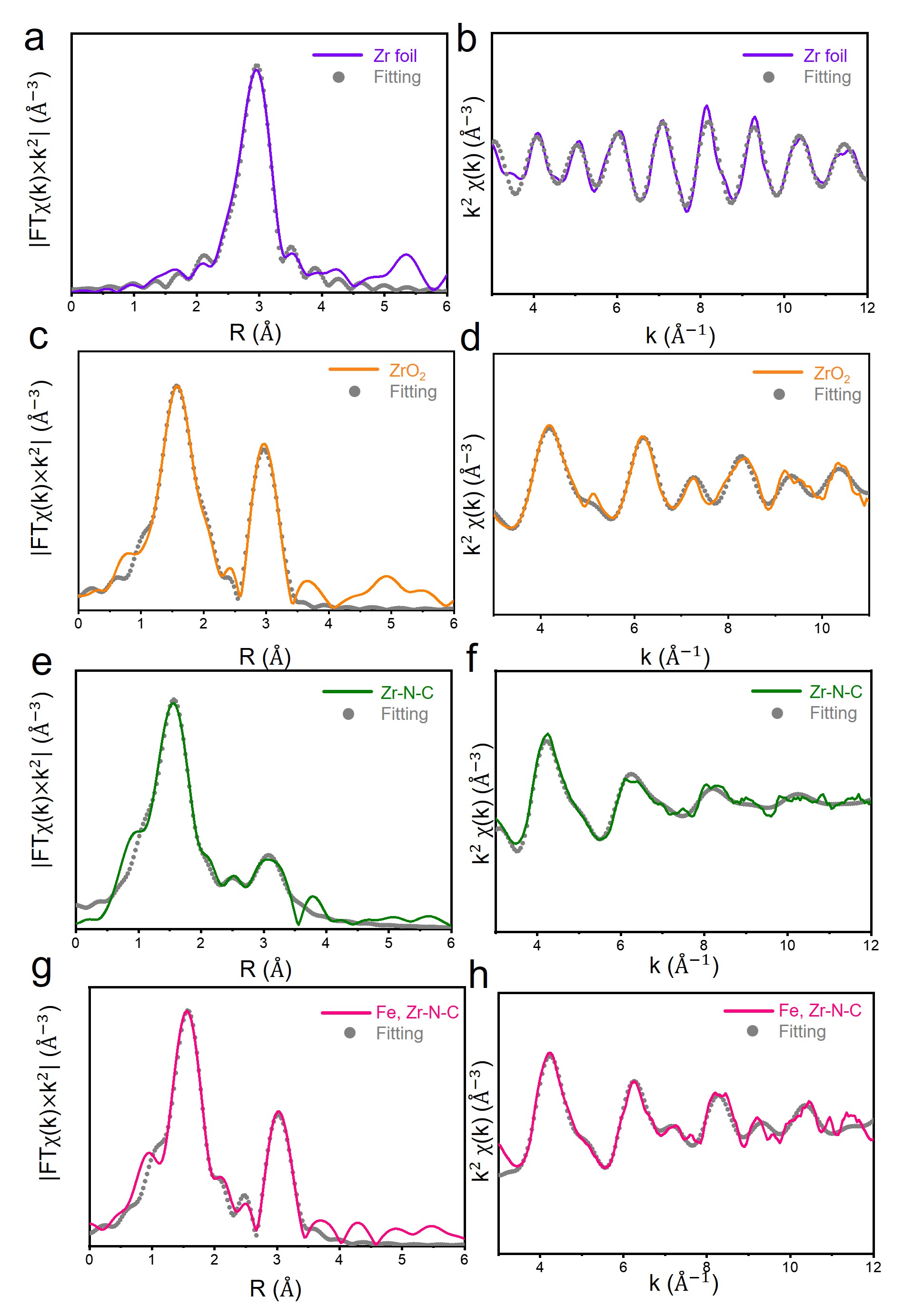


**Figure S15**. EXAFS spectral fitting for Zr foil (a) , ZrO_2_ (c), Zr-N-C(e) and Fe,Zr-N-C(g) in q space at Fe K-edge; EXAFS spectra fitting for Zr foil (b) , ZrO_2_ (d), Zr-N-C(f) and Fe,Zr-N-C(h)in k space at Fe K-edge.

**Table S5**. Structural parameters extracted from the Fe K-edge EXAFS fitting

| **Sample** | **Shell** | **CN** | **R(Å)** | **σ^2^(Å^2^)** | **R factor** |
| --- | --- | --- | --- | --- | --- |
| Fe foil | Fe-Fe | 8* | 2.47±0.01 | 0.0052±0.0004 | 0.0066 |
|  | Fe-Fe | 6* | 2.85±0.01 | 0.0053±0.0011 |  |
| FePc | Fe-N | 4.0±0.4 | 1.97±0.01 | 0.0054±0.0019 | 0.0082 |
| Fe_2_O_3_ | Fe-O | 3.7±0.4 | 1.98±0.01 | 0.0134±0.0018 | 0.0026 |
|  | Fe-Fe | 2.4±0.3 | 2.98±0.01 | 0.0078±0.0010 |  |
|  | Fe-Fe | 3.5±0.6 | 3.65±0.01 | 0.0007±0.0016 |  |
| Fe, Zr-N-C | Fe-N | 3.9±0.2 | 2.00±0.01 | 0.0086±0.0011 | 0.0044 |
|  | Fe-Zr | 1.2±0.4 | 2.75±0.01 | 0.0092±0.0028 |  |
|  | Fe-C | 2.6±0.5 | 2.93±0.01 | 0.0007±0.0018 | 0.0034 |
| Fe-N-C | Fe-N | 4.1±0.7 | 2.01±0.01 | 0.0094±0.0032 | 0.0158 |

**Table S6.** Structural parameters extracted from the Zr K-edge EXAFS fitting

| **Sample** | **Shell** | **CN** | **R(Å)** | **σ^2^(Å^2^)** | **R factor** |
| --- | --- | --- | --- | --- | --- |
| Zr foil | Zr-Zr | 12 | 3.22±0.01 | 0.0082±0.0006 | 0.0085 |
| ZrO_2_ | Zr-O | 3.8±0.4 | 2.11±0.01 | 0.0003±0.0014 | 0.0070 |
|  | Zr-O | 3.3±0.2 | 2.28±0.01 |  |  |
|  | Zr-(O)-Zr | 16.1±2.3 | 3.46±0.01 | 0.0133±0.0013 |  |
| Fe, Zr-N-C | Zr-N | 5.6±0.9 | 2.11±0.01 | 0.0006±0.0017 | 0.0101 |
|  | Zr-(N)-Fe | 3.4±0.7 | 3.30±0.01 | 0.0092±0.0028 |  |
| Zr-N-C | Zr-N | 5.1±0.6 | 2.11±0.01 | 0.0031±0.0013 | 0.0087 |

CN, coordination number; R, the distance between absorber and backscatter atoms; σ^2^, Debye-Waller factor, Debye-Waller factor to account for both thermal and structural disorders; R factor is used to value the goodness of the fitting.

Error bounds that characterize the structural parameters obtained by EXAFS spectroscopy were estimated as CN ± 20%; R ± 1%; σ^2^ ± 20%; ΔE_0_ ± 20%. A reasonable range of EXAFS fitting parameters: 0.700 < Ѕ02 < 1.000; CN > 0; σ^2^ > 0 Å2; |ΔE_0_| < 15 eV; R factor < 0.02.

**
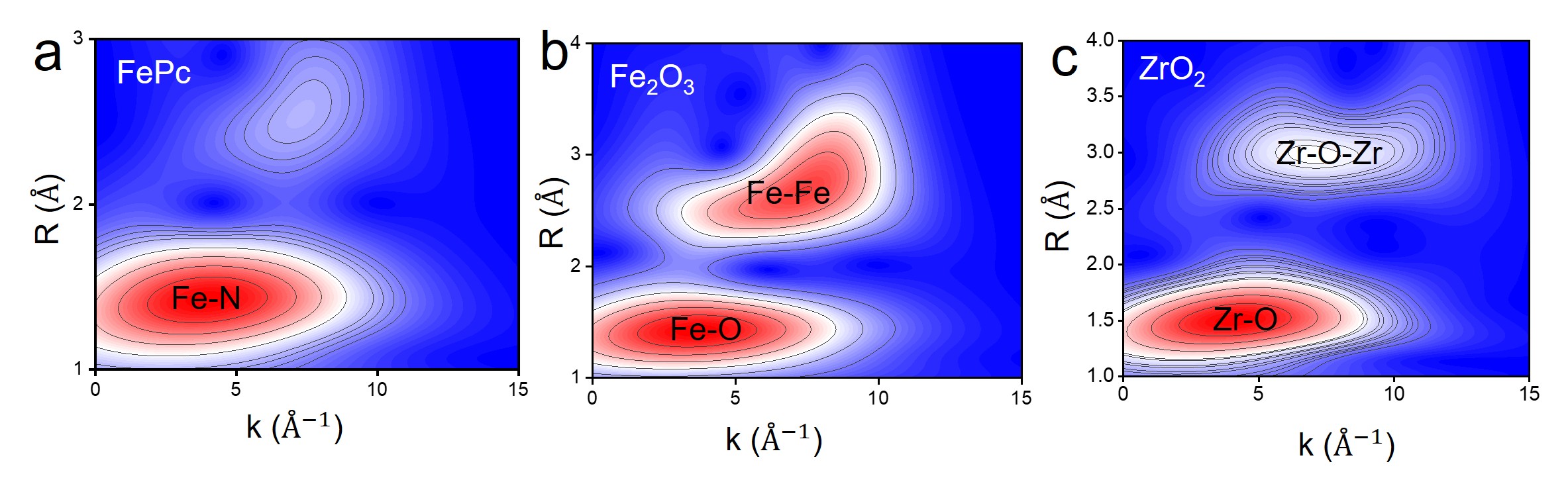
**

**Figure S16. a-b)** WT plots of FePc and Fe_2_O_3_; c) WT plots of ZrO_2_


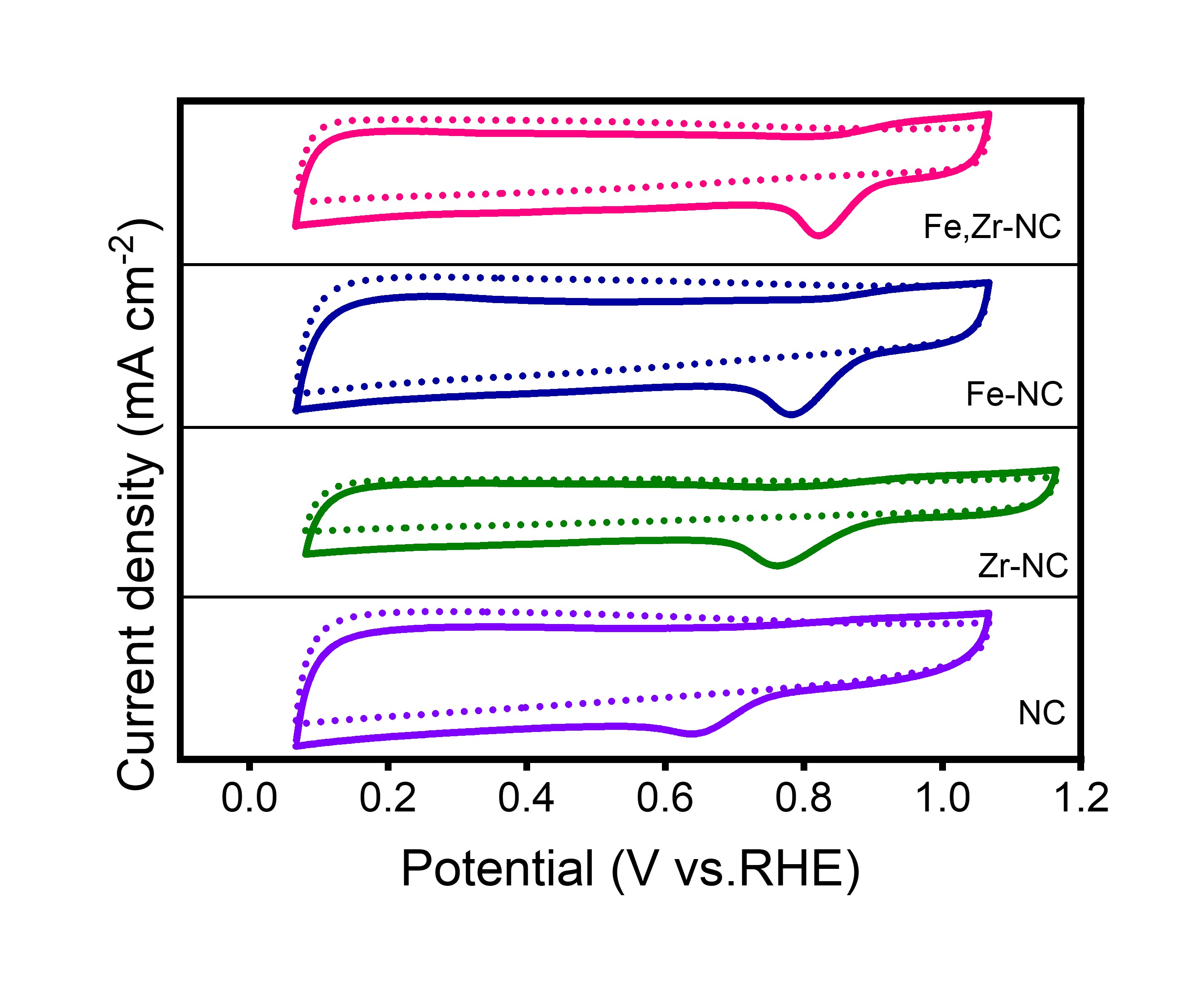


**Figure S17.** The CV curves of NC, Zr-NC, Fe-NC and Fe,Zr-NC in 0.1 M KOH.


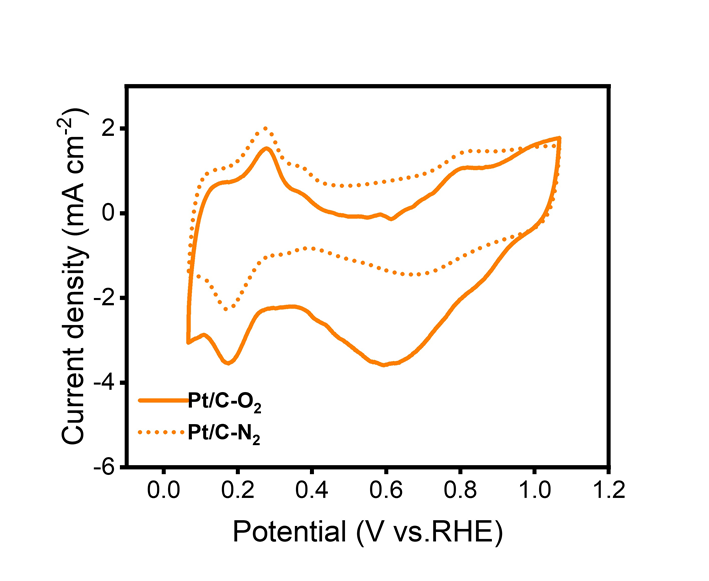


**Figure S18.** The CV curves of Pt/C in 0.1 M KOH.

**Table S7.** ORR performance comparison of Fe,Zr-NC and other reported state-of-the-art catalysts in alkaline electrolytes.

| Catalysts | Catalyst Loading  Density (mg cm^-2^) | E_onset_ (V) | E_1/2_ (V) | Reference |
| --- | --- | --- | --- | --- |
| **Fe,Zr-NC** | **0.202** | **1.06V** | **0.891** | **This Work** |
| 1MIL/40ZIF -1000 | 0.5 | 1 | 0.88 | ^[5]^ |
| Fe,Ni/NC@NG | - | 0.949 | 0.858 | ^[6]^ |
| FeNiCo@NC-P | - | 1.55 | 0.84 | ^[7]^ |
| Fe-N/P-C-700 | 0.6 | 0.941 | 0.867 | ^[8]^ |
| CoNi-SAs/NC | 0.5 | 0.88 | 0.76 | ^[9]^ |
| p-Fe-N-CNFs | 0.6 | 0.94 | 0.82 | ^[10]^ |
| CoNi@NCNT/NT | - | 1 | 0.87 | ^[11]^ |
| Fe/SNC | 0.51 | <1 | 0.86 | ^[12]^ |
| Fe-N-C (50 nm) | 0.5 | 0.95 | 0.81 | ^[13]^ |
| 3Fe-N-C HNSs | 0.255 | 1.045 | 0.87 | ^[14]^ |
| FeNC-S-FexC/Fe_0.1_ | 0.1 | 1.05 | 0.873 | ^[15]^ |
| Zn-N-C-1 | 0.5 | - | 0.873 | ^[16]^ |
| sFe/OES | 0.4 | 1 | 0.85 | ^[17]^ |
| PtSA–PtCo NCs/N–CNTs-900 | 0.26 | 1.48 | 0.86 | ^[18]^ |
| A-MnO2/NSPC-2 | 0.29 | 1.51 | 0.87 | ^[19]^ |
| Fe 1 -HNC -500 -850 | 0.2 | 0.93 | 0.842 | ^[20]^ |

**Table S8.** TOF values at 0.8 V for different catalysts.

| **Sample** | **TOF (s^-1^)** |
| --- | --- |
| Fe,Zr-NC | 16.28 |
| Fe -NC | 2.60 |
| Zr-NC | 0.87 |


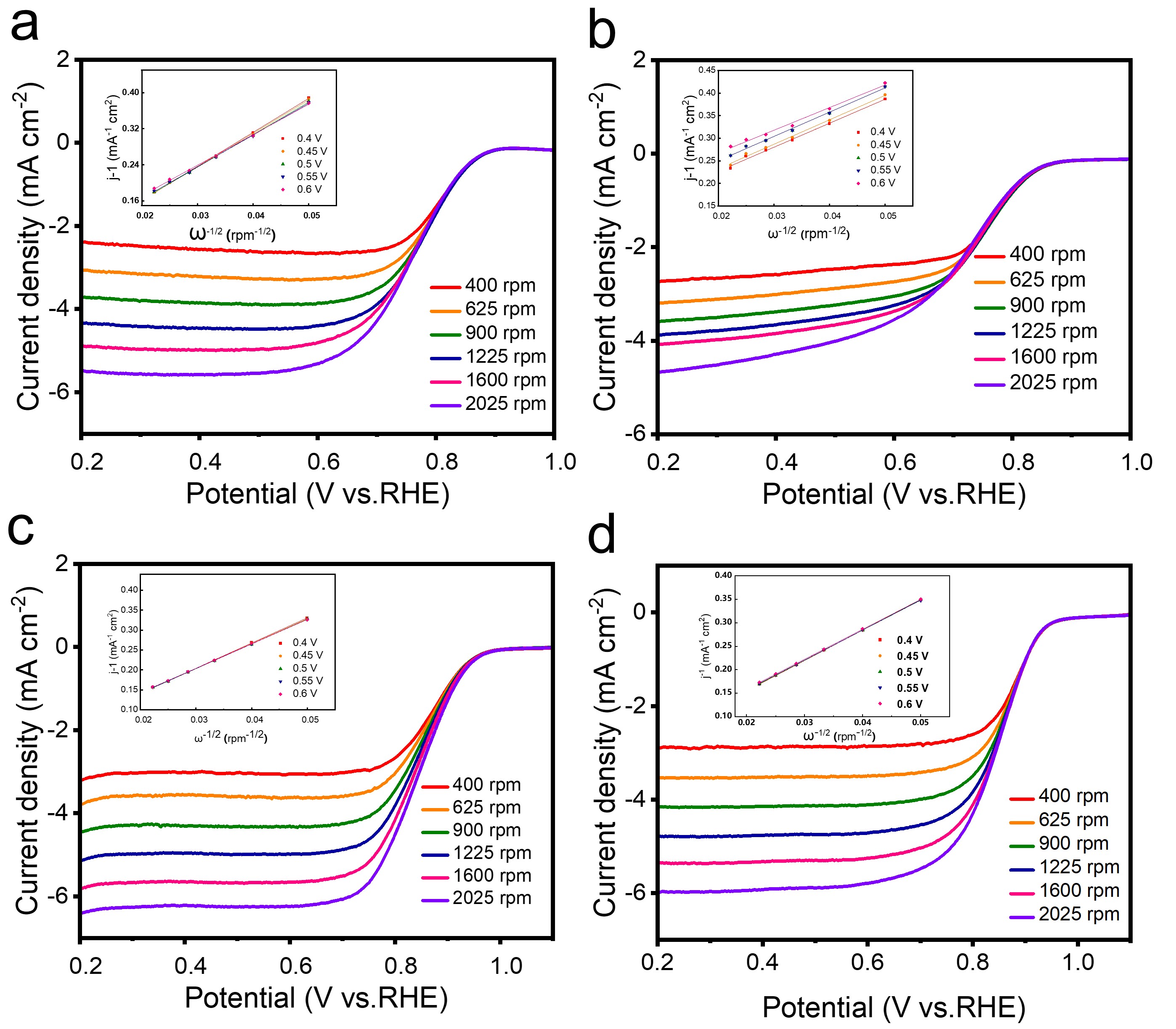


**Figure S19.** LSV polarization curves at various rotation rates and corresponding K-L plots of a) Zr-NC, b) NC, c) PT/C, and d) Fe-NC in 0.1 M KOH.


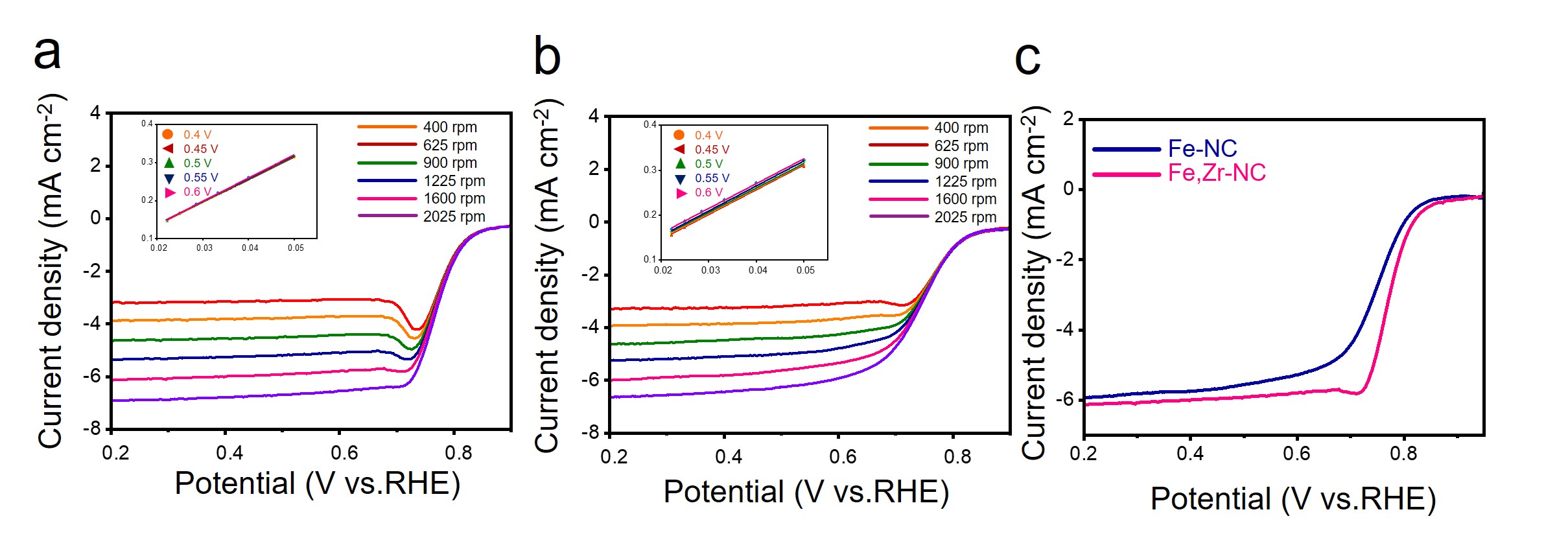


**Figure S20.** LSV polarization curves at various rotation rates and corresponding K-L plots of a) Fe,Zr-NC, b) Fe-NC in 0.1 M HClO_4_. c) LSV curves of Fe,Zr-NC and Fe-NC in 0.1 M HClO_4_ at 1600 rpm.

In order to further prove that the catalytic activity of Fe,Zr-NC is better than that of Fe-NC in acid electrolyte, the LSV tests was conducted in 0.1 M HClO_4_ electrolyte, and the results showed that Fe,Zr-NC (*E*_1/2_=0.77 V vs.RHE) still has better electrochemical activity than Fe-NC (*E*_1/2_=0.74 V vs.RHE) under acidic conditions.


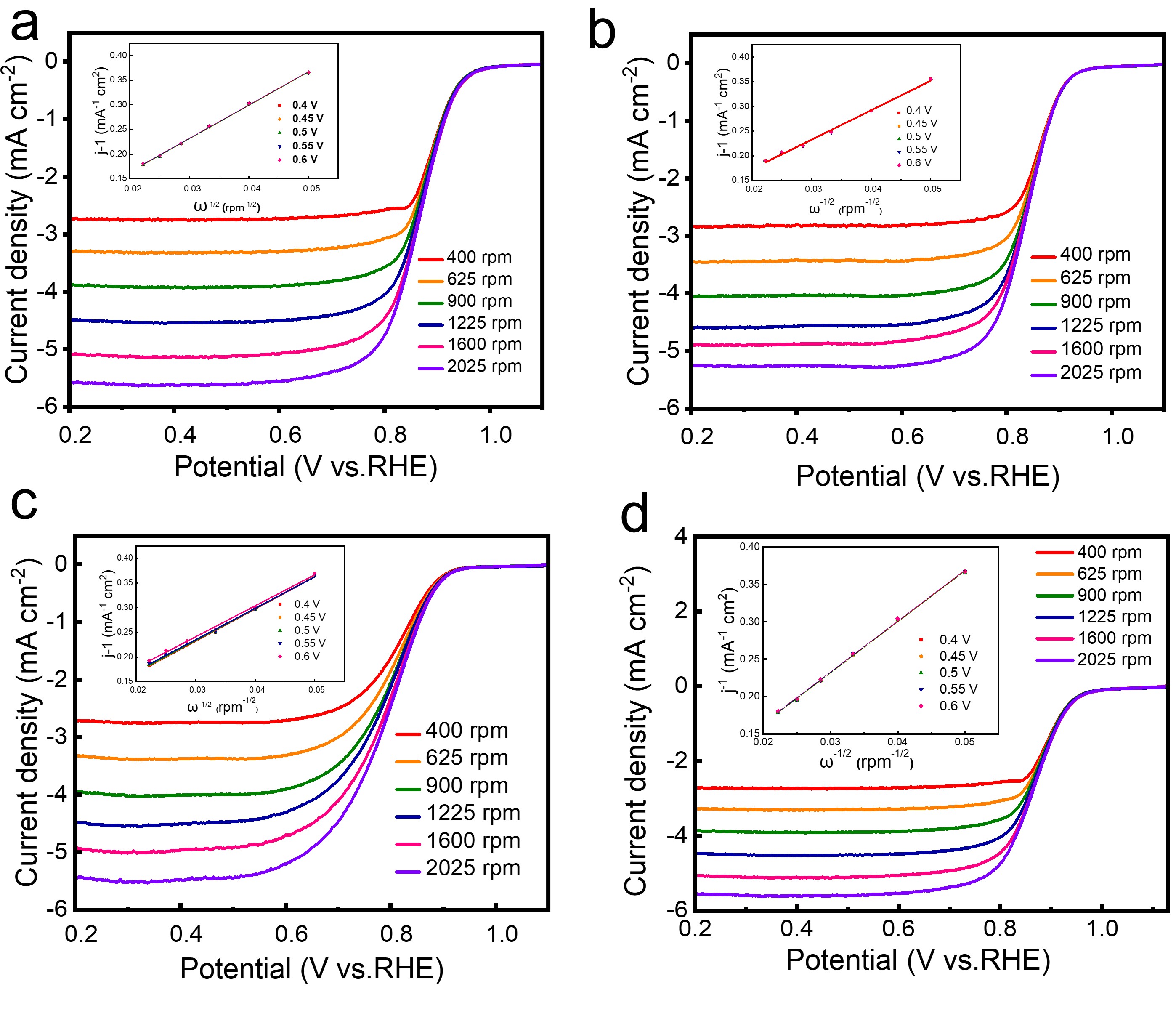


**Figure S21.** LSV polarization curves at various rotation rates and corresponding K-L plots of a) Fe_1_Zr_1_-NC-950, b) Fe_1.5_Zr_1_-NC-900, c) Fe_1.5_Zr_1_-NC-1000, and d) Fe_2_Zr_1_-NC-950 in 0.1 M KOH.

As shown in Figure S19, the electron transfer number during the oxygen reduction process for catalysts synthesized at different temperatures and Fe/Zr ratios was calculated using the Koutecky-Levich (K-L) equation, yielding values in the range of 3.8–4.2, indicating the retention of a four-electron transfer pathway.


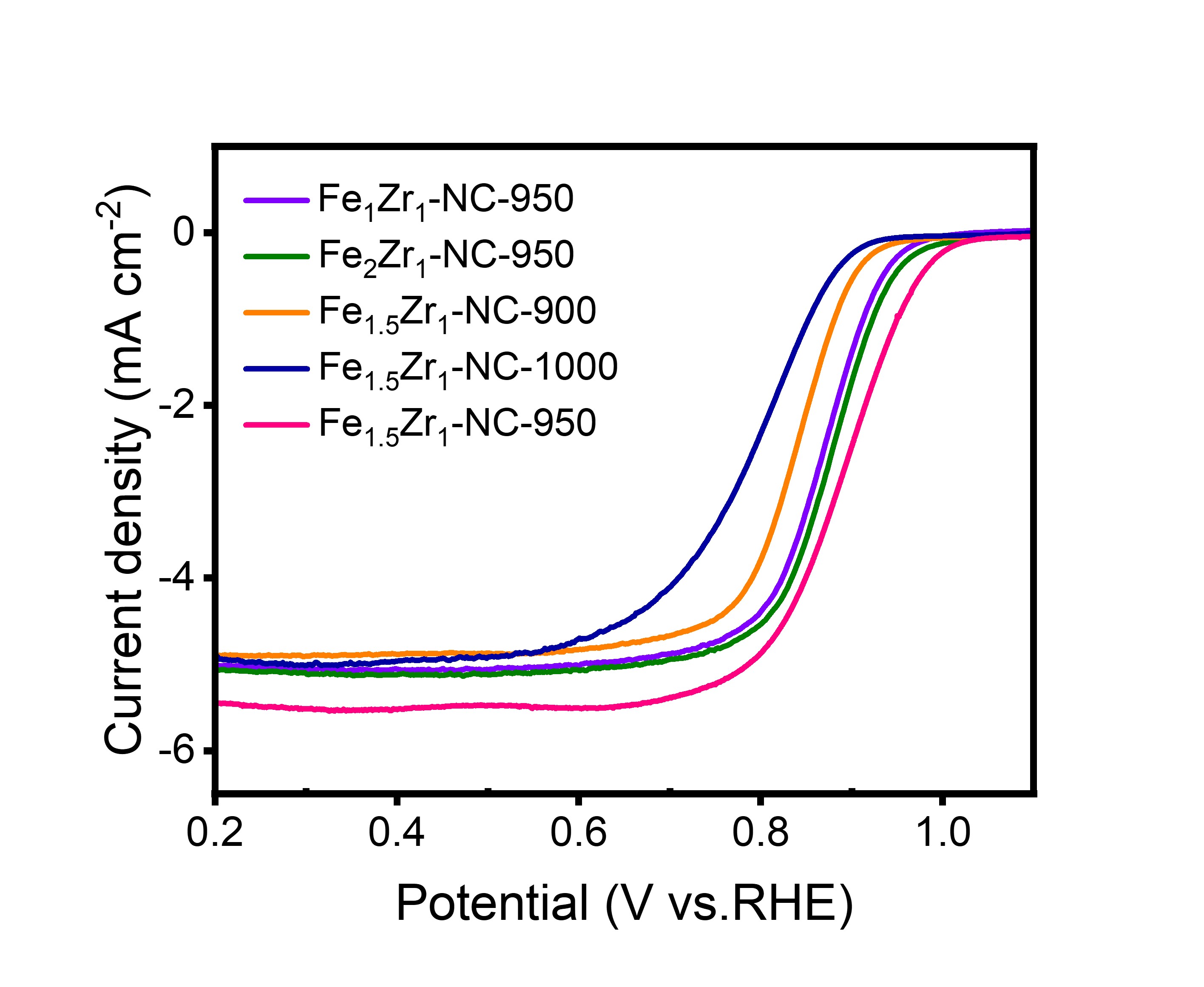


**Figure S22.** LSV curves of Fe_1_Zr_1_-NC-950, Fe_1.5_Zr_1_-NC-900, Fe_1.5_Zr_1_-NC-1000, Fe_2_Zr_1_-NC-950 and Fe_1.5_Zr_1_-NC-950 in 0.1 M KOH at1600 rpm.

In order to obtain the optimal temperature and the optimal temperature, we also chose the insulation temperature of 900 ℃ and 1000℃ when Fe:Zr=1.5:1, and when the optimal temperature is determined to be 950 ℃, the ratio of Fe to Zr is 1:1 and 2:1, respectively. The results obtained by testing the appeal sample are shown in Figure S20. The half-wave potentials of Fe_1_Zr_1_-N-C-950, Fe_2_Zr_1_-N-C-950, Fe_1.5_Zr_1_-N-C-900, and Fe_1.5_Zr_1_-N-C-1000 catalysts were measured as 0.869 V vs. RHE, 0.867 V vs. RHE, 0.841 V vs. RHE, and 0.791 V vs. RHE, respectively. Notably, all values were lower than that of the Fe_1.5_Zr_1_-N-C-950 catalyst, indicating its superior electrocatalytic performance under the optimized Fe: Zr ratio and carbonization temperature.


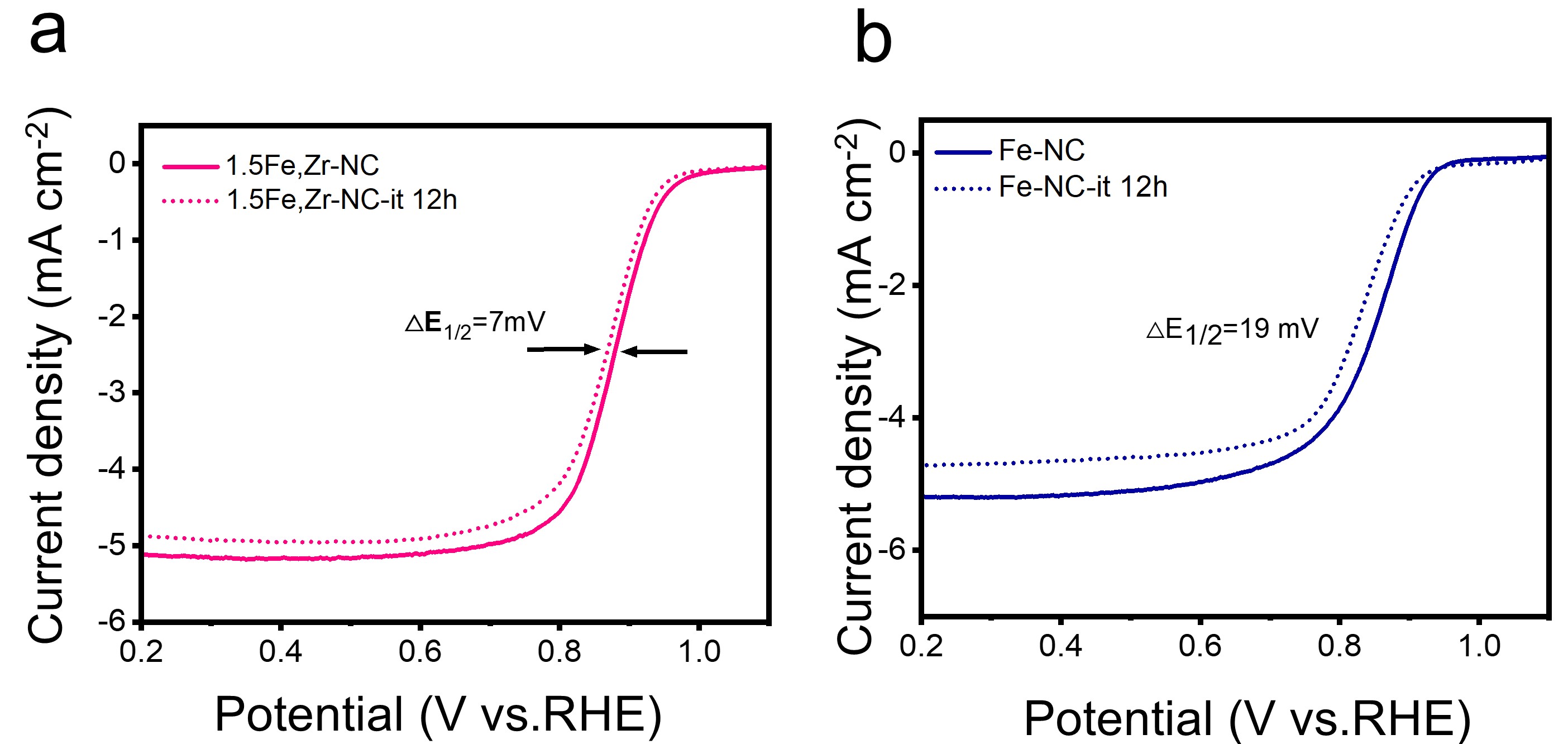


**Figure S23.** Comparison of LSV before and after galvanostat stability test of a)Fe, Zr-NC and b)Fe-NC


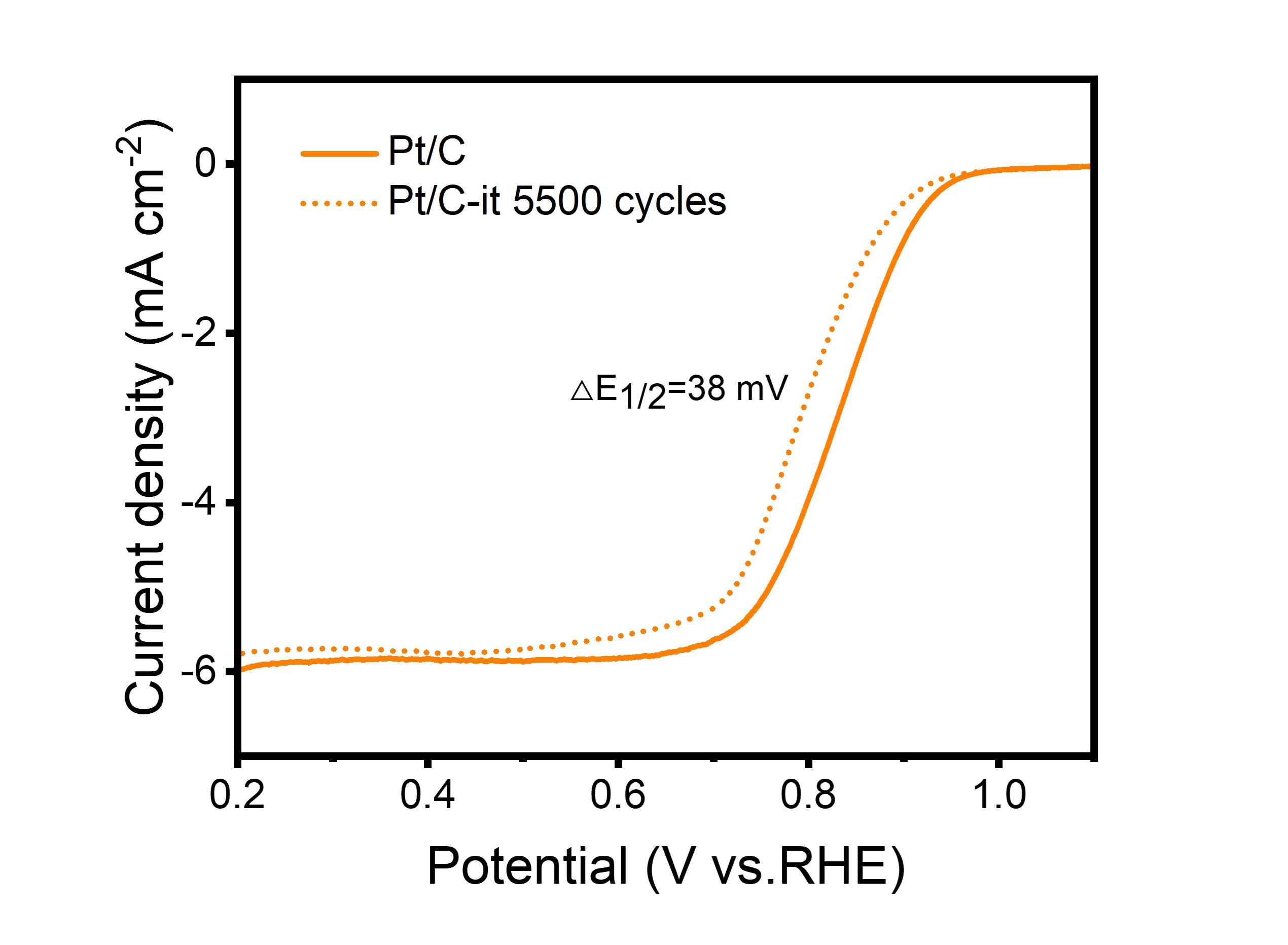


**Figure S24.** LSV curves of Pt/C before and after 5500 CV cycles.


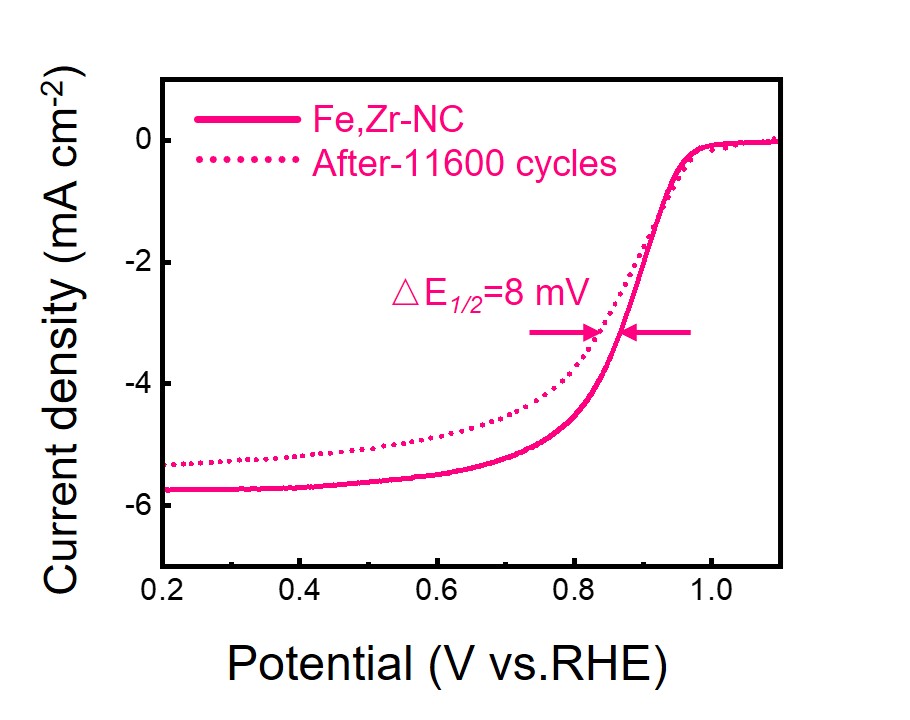


**Figure S25.** LSV curves of Fe,Zr-NC before and after 11500 CV cycles

**Table S9.** Performance comparison of Fe,Zr-NC and other non-precious metal catalysts in aqueous Zn-air batteries with an alkaline electrolyte.

| Catalysts | Catalyst Loading  Density  (mg cm^-2^) | Peak Power  Density (mW cm^-2^) | Specific  capacity (mAhgZn^-1^) | Cycling time (h)  @current density  (mA cm^-2^) | Ref. |
| --- | --- | --- | --- | --- | --- |
| **Fe,Zr-NC** | **1** | **185.7** |  | **453@10** | **This Work** |
| Fe,Ni/NC@NG | - | 110 |  | 300@5 | ^[6]^ |
| PtSA–PtCo NCs/N–CNTs-900 | 1 | 110.6 | 794.6 | 220@10 | ^[18]^ |
| CoNi-SAs/NC | 1.4 | 101.4 | 750.9 | <100@5 | ^[9]^ |
| A-MnO_2_/NSPC-_2_ | 1 | 181 | 816 | 287@10 | ^[19]^ |
| NiFe@C@Co CNFs | - | 130 | 694 | 200@10 | ^[21]^ |
| N2-NiFe-PBA/NCF/CC -60 | - | 155 | 775 | 330@10 | ^[22]^ |
| Ni_x_Co_1_–x@Ni_x_Co_1_  –xO/NCNT | - | 157 | 746 | 30@10 | ^[23]^ |
| FeNC-S-FexC/Fe | 0.2 | 149 | - | 380@2 | ^[15]^ |
| Fe-N/P-C-700 | 3 | 133 | - | 40 | ^[8]^ |
| Co_3_FeS_1.5_(OH)_6_ | 0.5 | 113.1 | - | 108@2 | ^[24]^ |
| NiCo2S4@gC_3_N_4_-CNT | 2.33 | 142 | 458.7 | >100@10 | ^[25]^ |

**
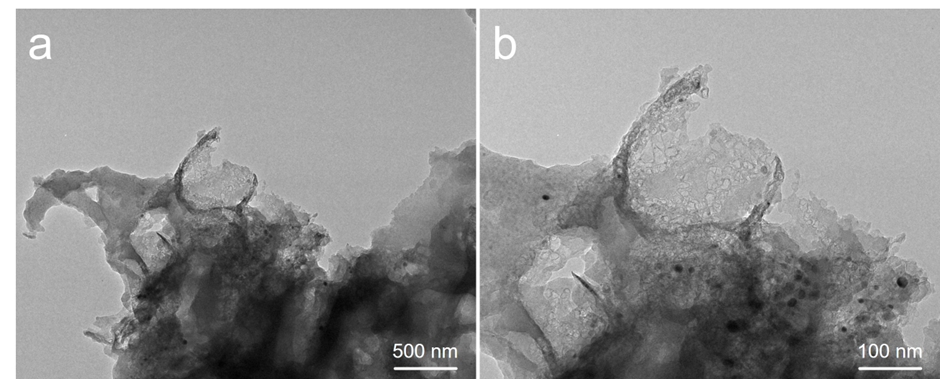
**

**Figure S26.**TEM image of Fe -NC after tested for battery stability for 170h.

**
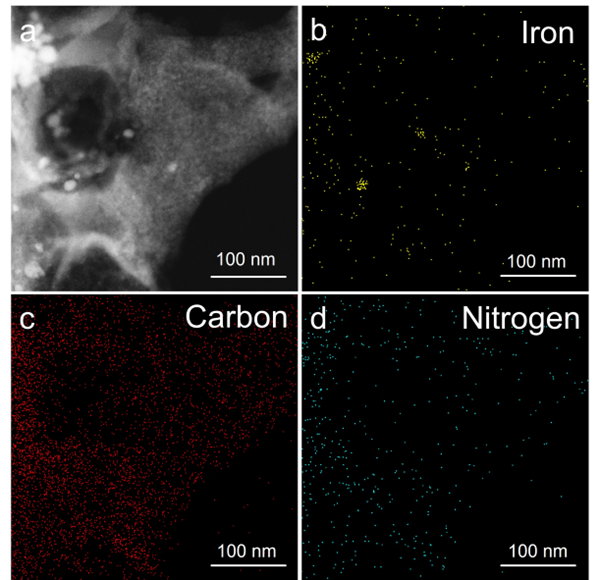
**

**Figure S27.** a)TEM and b-d) mapping images of Fe-NC after tested for battery stability for 170h.


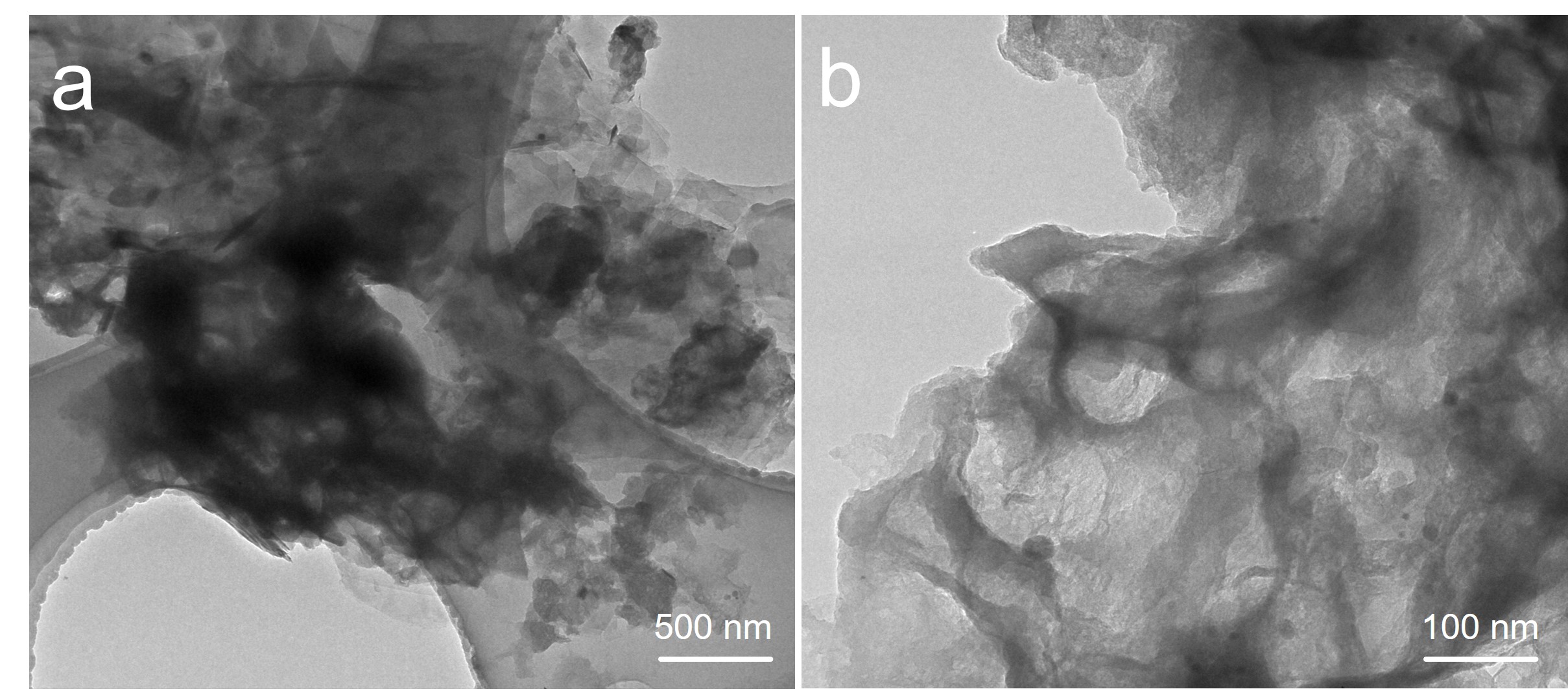


**Figure S28.**TEM image of Fe,Zr-NC after 453h tests for battery stability.


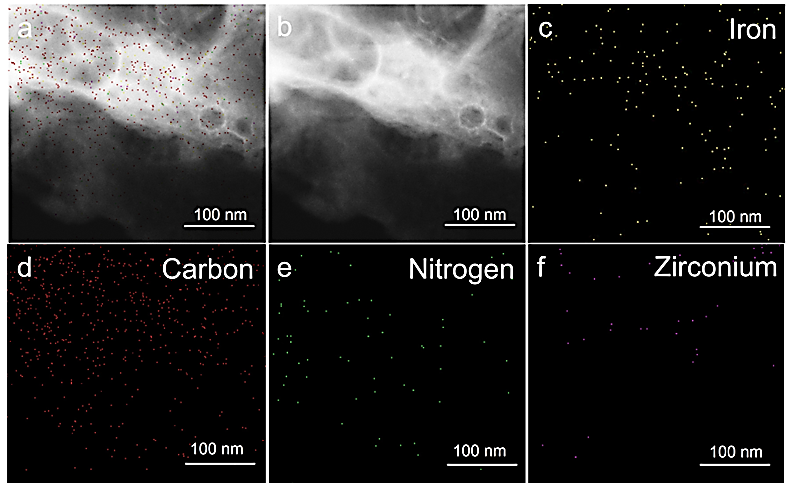


**Figure S29.** a) TEM and b-f) mapping images of Fe, Zr-NC after tested for battery stability for 453h.


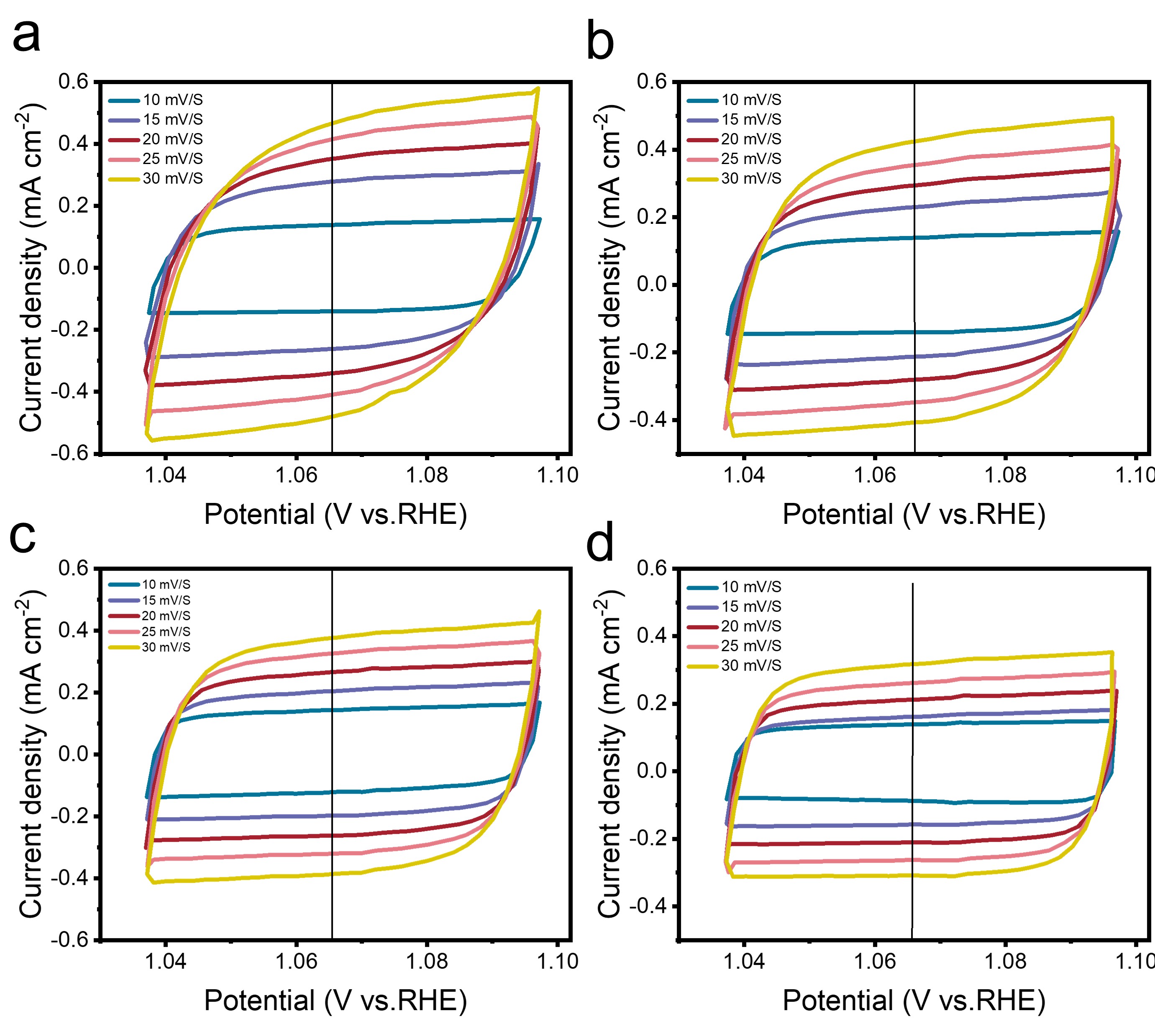


**Figure S30.** CV curves of a) Fe,Zr-NC before i-t test, b) Fe,Zr-NC after 12 hours of i-t test, c) Fe-NC before i-t test, and d) Fe-NC after 12 hours of i-t test at different scan rates ranging from 10 to 30 mV s^−1^ with a step of 5 mV s^−1^ in 0.1 M KOH.


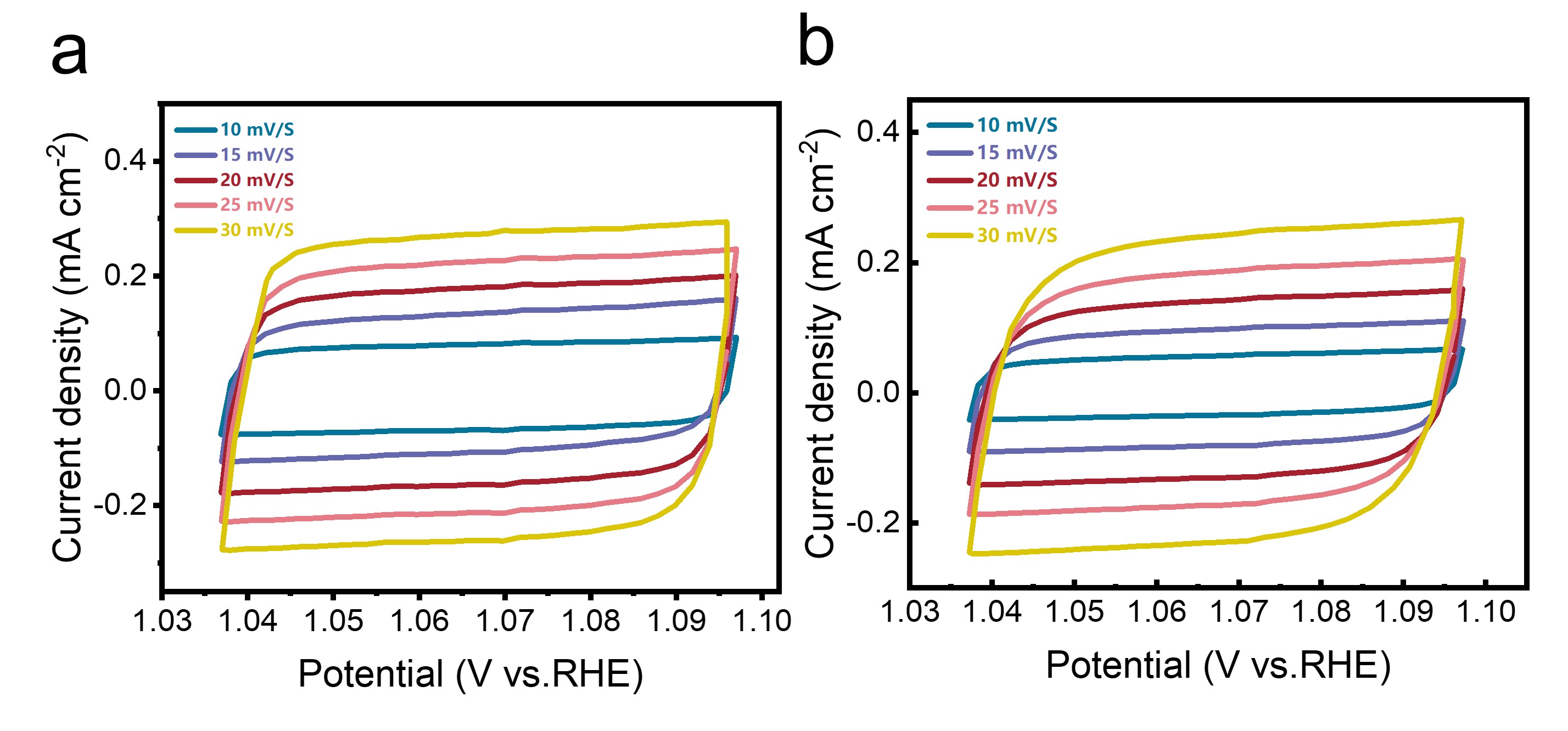


**Figure S31.** CV curves of a) Pt/C before i-t test, b) Pt/C after 12 hours of i-t test, at different scan rates ranging from 10 to 30 mV s^−1^ with a step of 5 mV s^−1^ in 0.1 M KOH


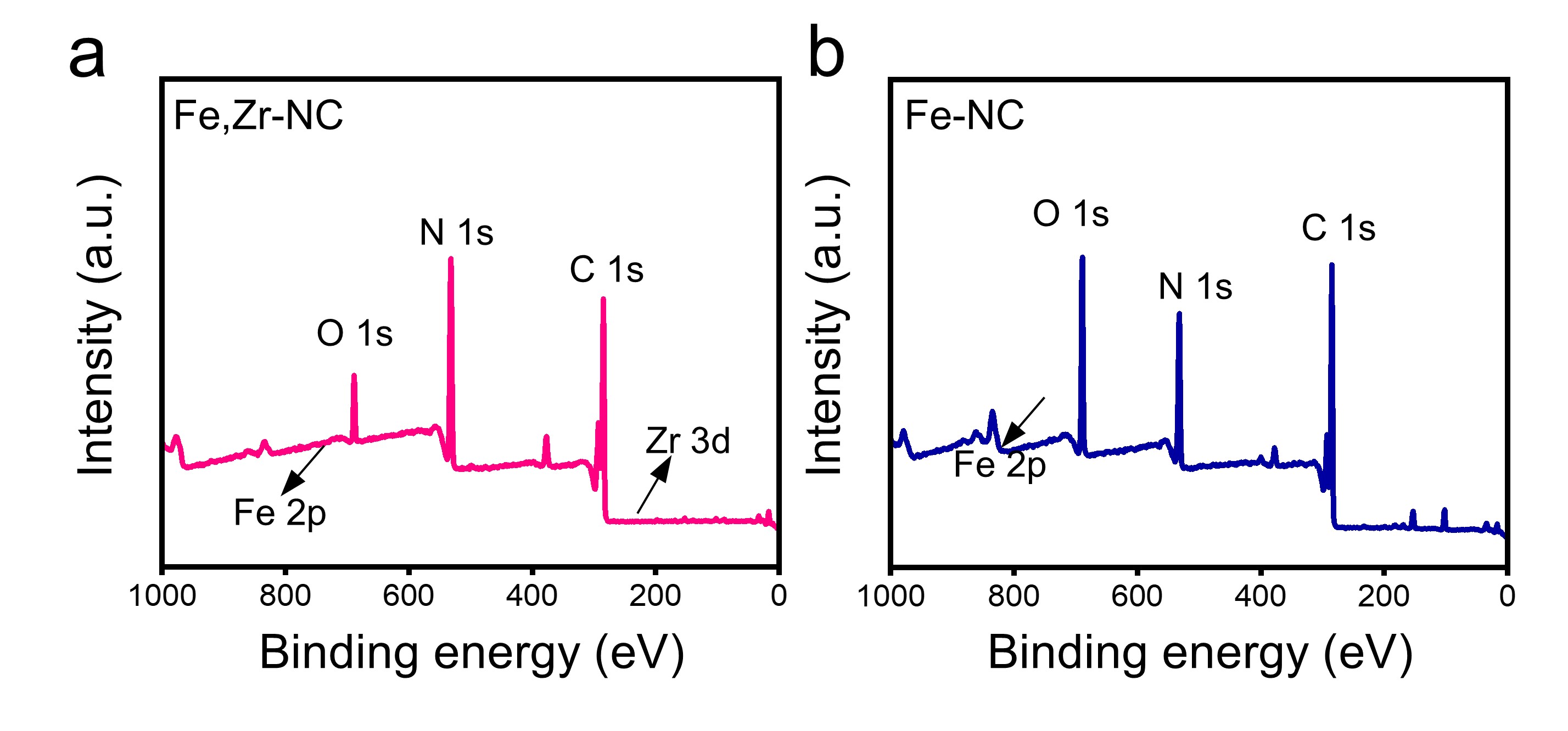


**Figure S32.** XPS survey spectra of a) Fe,Zr-NC and b)Fe-NC after 5500 CV cycles


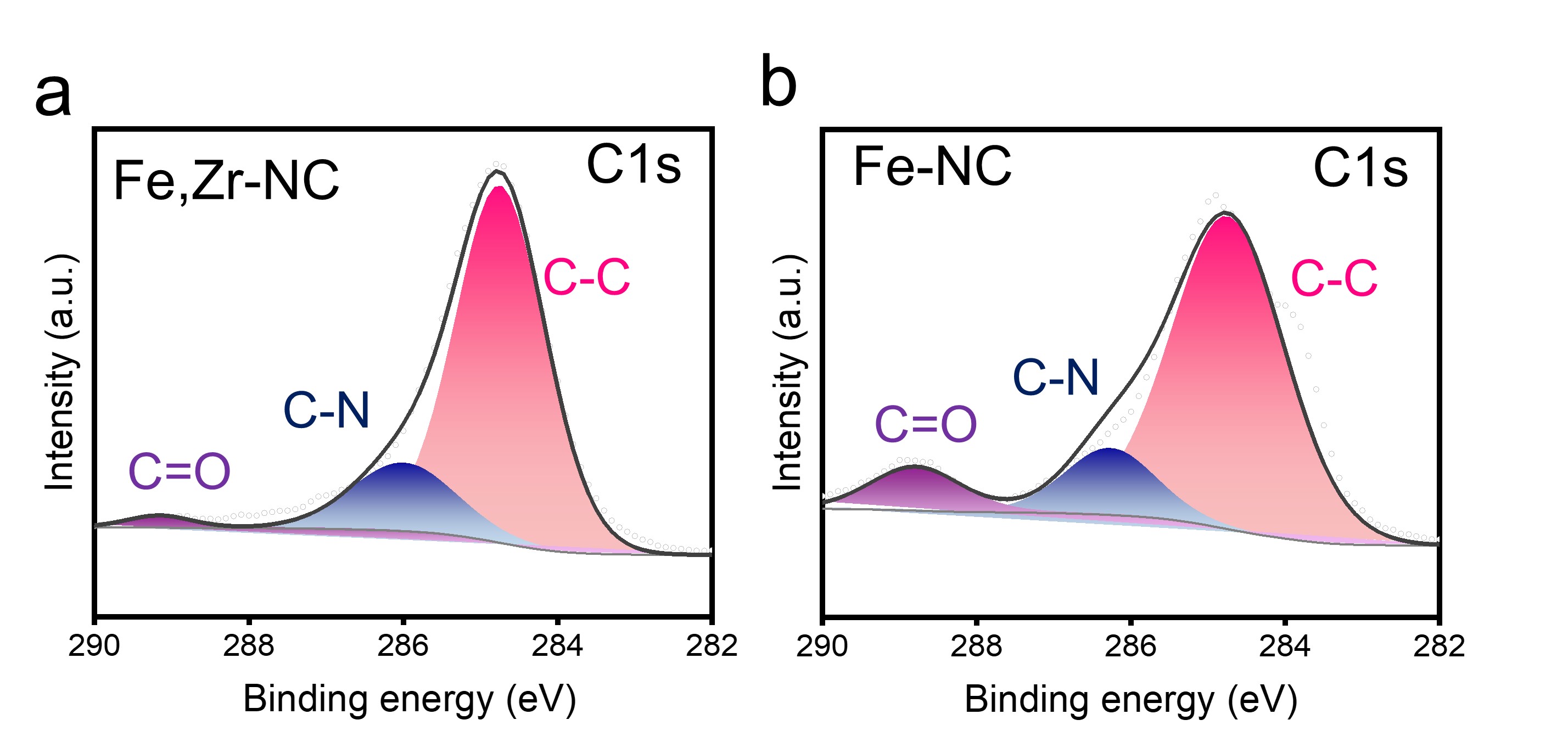


**Figure S33.** High-resolution XPS C1*s* spectra of a) Fe,Zr-NC and b)Fe -NC after the 5500 CV cycles

stability test.


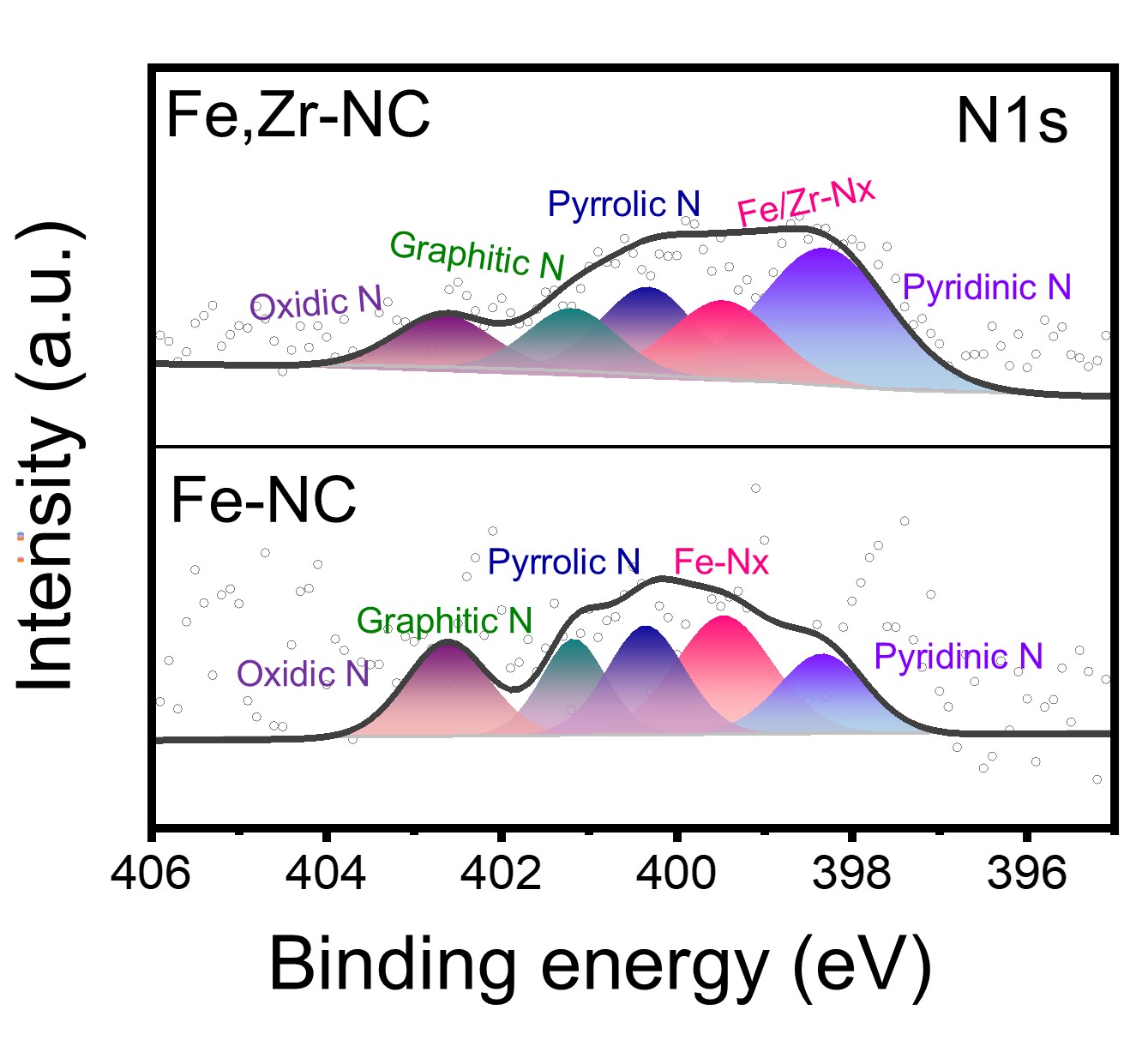


**Figure S34.** High-resolution XPS N 1*s* spectra of Fe,Zr-NC and Fe -NC after the 5500 CV cycles

stability test.


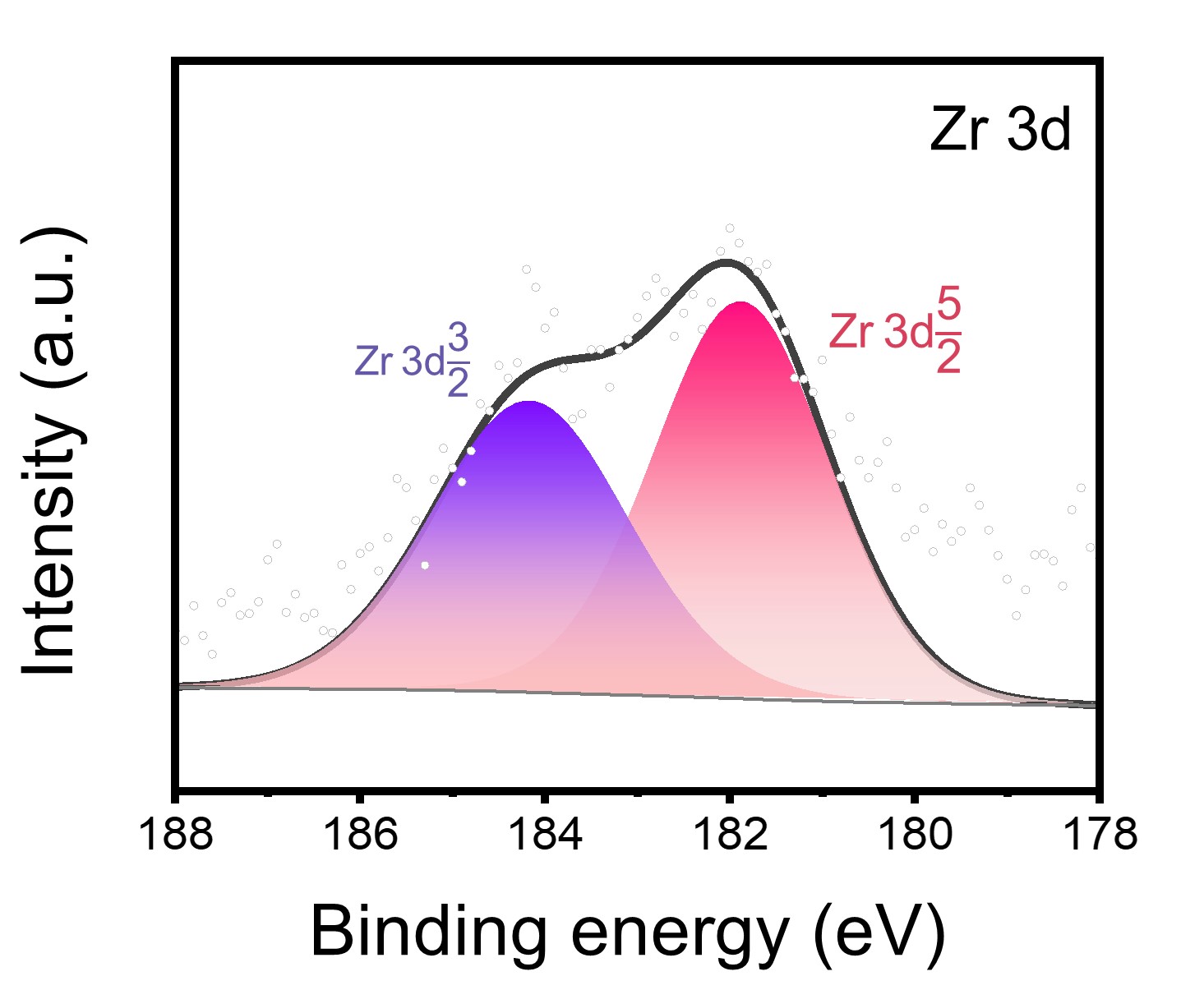


**Figure S35.** High-resolution XPS Zr 3d spectra of Fe,Zr-NC after the 5500 CV cycles stability test.

**Table S10.** Stabilizing various nitrogen levels after testing.

| Sample | Oxidized-N | Graphitic-N | Pyrrolic-N | M-N | Pyridinic-N |
| --- | --- | --- | --- | --- | --- |
| Fe,Zr-NC | 11.8 | 12.5 | 18.4 | 18.1 | 39.2 |
| Fe-NC | 19.6 | 15.1 | 20.7 | 27.9 | 16.6 |


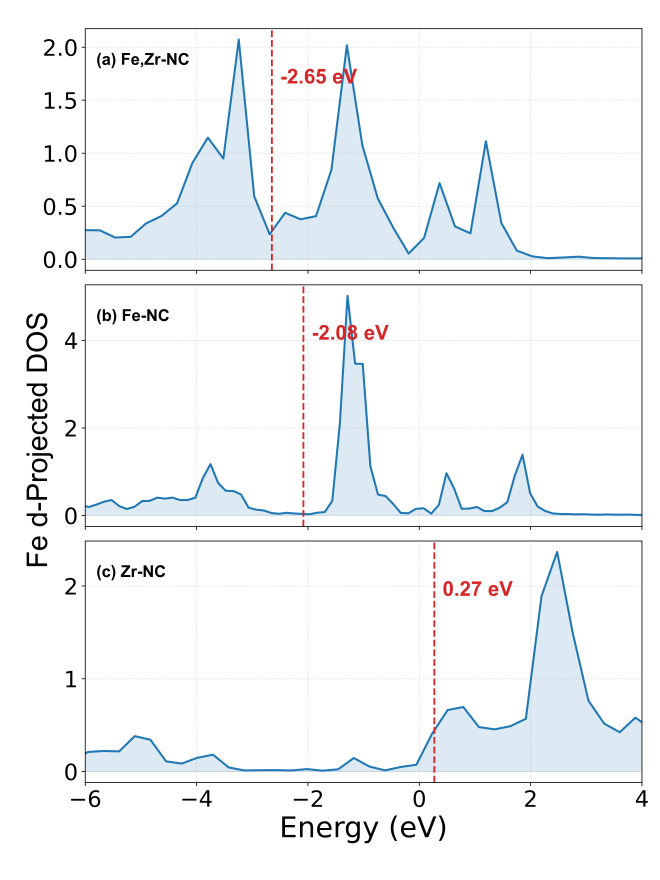


**Figure S36.** Projected density of states (PDOS) and corresponding d-band centers of (a) Fe in Fe,Zr-NC, (b) Fe in Fe-NC, and (c) Zr in Zr-NC. The Fermi level is set to 0 eV. Compared to Fe-NC (-2.08 eV), the Fe d-band center in Fe,Zr-NC (-2.65 eV) shifts downward, indicating weakened Fe-O interaction, which is consistent with the reduced *OH adsorption energy.


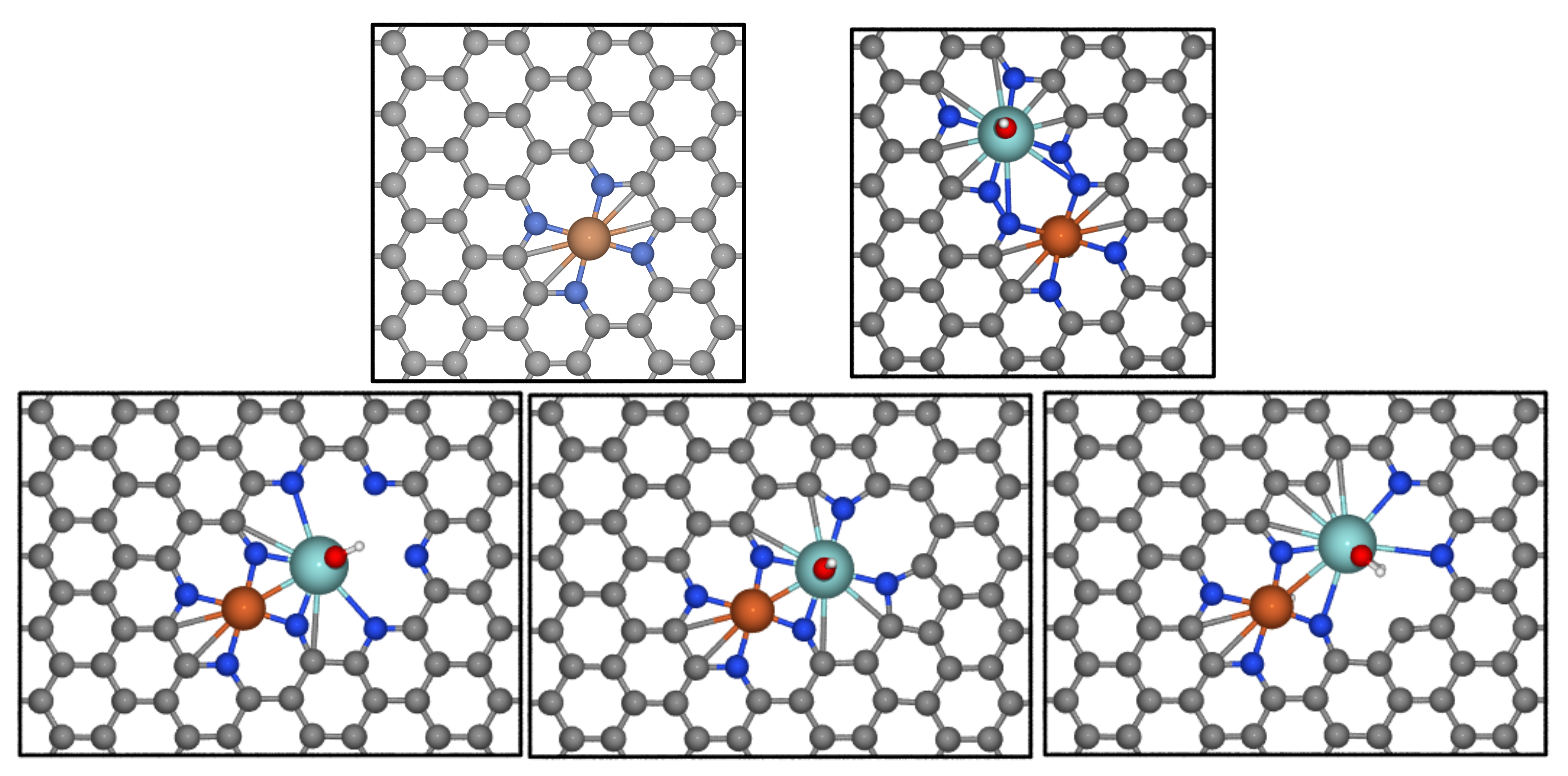


**Figure S37.** DFT simulation cells for Fe-NC, Fe,Zr-NC. Gray: carbon, Red:oxygen; Blue: nitrogen; Orange; Fe; Cyan: Zr; White: hydrogen.

**References**

[1] a)J. J. Mortensen, L. B. Hansen, K. W. Jacobsen, *Phys. Rev. B* **2005**, 71, 035109; b)A. Hjorth Larsen, J. Jørgen Mortensen, J. Blomqvist, I. E. Castelli, R. Christensen, M. Dułak, J. Friis, M. N. Groves, B. Hammer, C. Hargus, E. D. Hermes, P. C. Jennings, P. Bjerre Jensen, J. Kermode, J. R. Kitchin, E. Leonhard Kolsbjerg, J. Kubal, K. Kaasbjerg, S. Lysgaard, J. Bergmann Maronsson, T. Maxson, T. Olsen, L. Pastewka, A. Peterson, C. Rostgaard, J. Schiøtz, O. Schütt, M. Strange, K. S. Thygesen, T. Vegge, L. Vilhelmsen, M. Walter, Z. Zeng, K. W. Jacobsen, *J Phys Condens Matter* **2017**, 29, 273002; c)S. R. Bahn, K. W. Jacobsen, *Computing in Science & Engineering* **2002**, 4, 56.

[2] J. Wellendorff, K. T. Lundgaard, A. Møgelhøj, V. Petzold, D. D. Landis, J. K. Nørskov, T. Bligaard, K. W. Jacobsen, *Phys. Rev. B* **2012**, 85, 235149.

[3] J. K. Nørskov, J. Rossmeisl, A. Logadottir, L. Lindqvist, J. R. Kitchin, T. Bligaard, H. Jónsson, *The J. Phys. Chem. B* **2004**, 108, 17886.

[4] F. Calle-Vallejo, J. I. Martínez, J. Rossmeisl, *Phys. Chem. Chem. Phys.* **2011**, 13, 15639.

[5] H. Wang, F.-X. Yin, N. Liu, R.-H. Kou, X.-B. He, C.-J. Sun, B.-H. Chen, D.-J. Liu, H.-Q. Yin, *Adv. Funct. Mater.* **2019**, 29, 1901531.

[6] Q. Jing, Z. Mei, X. Sheng, X. Zou, Q. Xu, L. Wang, H. Guo, *Adv. Funct. Mater.* **2024**, 34, 2307002.

[7] D. Ren, J. Ying, M. Xiao, Y.-P. Deng, J. Ou, J. Zhu, G. Liu, Y. Pei, S. Li, A. M. Jauhar, H. Jin, S. Wang, D. Su, A. Yu, Z. Chen, *Adv. Funct. Mater.* **2020**, 30, 1908167.

[8] K. Yuan, D. Lützenkirchen-Hecht, L. Li, L. Shuai, Y. Li, R. Cao, M. Qiu, X. Zhuang, M. K. H. Leung, Y. Chen, U. Scherf, *J. Am. Chem. Soc.* **2020**, 142, 2404.

[9] X. Han, X. Ling, D. Yu, D. Xie, L. Li, S. Peng, C. Zhong, N. Zhao, Y. Deng, W. Hu, **2019**, 31, 1905622.

[10] B.-C. Hu, Z.-Y. Wu, S.-Q. Chu, H.-W. Zhu, H.-W. Liang, J. Zhang, S.-H. Yu, *Energy & Environmental Science* **2018**, 11, 2208.

[11] W. Niu, S. Pakhira, K. Marcus, Z. Li, J. L. Mendoza-Cortes, Y. Yang, **2018**, 8, 1800480.

[12] H. Shen, E. Gracia-Espino, J. Ma, K. Zang, J. Luo, L. Wang, S. Gao, X. Mamat, G. Hu, T. Wagberg, S. Guo, *Adv. Mater.* **2017**, 56, 13800.

[13] C. Zhang, Y.-C. Wang, B. An, R. Huang, C. Wang, Z. Zhou, W. Lin, *Adv. Mater.* **2017**, 29, 1604556.

[14] Y. Chen, Z. Li, Y. Zhu, D. Sun, X. Liu, L. Xu, Y. Tang, *Adv. Mater.* **2019**, 31, 1806312.

[15] Y. Qiao, P. Yuan, Y. Hu, J. Zhang, S. Mu, J. Zhou, H. Li, H. Xia, J. He, Q. Xu, *Adv. Mater.* **2018**, 30, 1804504.

[16] J. Li, S. Chen, N. Yang, M. Deng, S. Ibraheem, J. Deng, J. Li, L. Li, Z. Wei, *Angew. Chem. Int. Ed.* **2019**, 58, 7035.

[17] C.-C. Hou, L. Zou, L. Sun, K. Zhang, Z. Liu, Y. Li, C. Li, R. Zou, J. Yu, Q. Xu, *Angew. Chem. Int. Ed.* **2020**, 59, 7384.

[18] W. Chen, X. Zhu, W. Wei, H. Chen, T. Dong, R. Wang, M. Liu, K. Ostrikov, P. Peng, S.-Q. Zang, *Small* **2023**, 19, 2304294.

[19] L. Huo, M. Lv, M. Li, X. Ni, J. Guan, J. Liu, S. Mei, Y. Yang, M. Zhu, Q. Feng, P. Geng, J. Hou, N. Huang, W. Liu, X. Y. Kong, Y. Zheng, L. Ye, *Adv. Mater.* **2024**, 36, 2312868.

[20] X. Zhang, S. Zhang, Y. Yang, L. Wang, Z. Mu, H. Zhu, X. Zhu, H. Xing, H. Xia, B. Huang, J. Li, S. Guo, E. Wang, *Adv. Mater.* **2020**, 32, 1906905.

[21] X. Chen, J. Pu, X. Hu, Y. Yao, Y. Dou, J. Jiang, W. Zhang, *Small* **2022**, 18, 2200578.

[22] C. Lai, H. Li, Y. Sheng, M. Zhou, W. Wang, M. Gong, K. Wang, K. Jiang, *Adv. Sci.* **2022**, 9, 2105925.

[23] R. Jena, S. Bhattacharyya, N. Bothra, V. Kashyap, S. K. Pati, T. K. Maji, *ACS Appl. Mater. Interf.* **2023**, 15, 27893.

[24] H.-F. Wang, C. Tang, B. Wang, B.-Q. Li, Q. Zhang, *Adv. Mater.* **2017**, 29, 1702327.

[25] X. Han, W. Zhang, X. Ma, C. Zhong, N. Zhao, W. Hu, Y. Deng, *Adv. Mater.* **2019**, 31, 1808281.
